# Cycles of light and dark co-ordinate reversible colony differentiation in *Listeria monocytogenes*

**DOI:** 10.1111/mmi.12140

**Published:** 2013-01-21

**Authors:** Teresa Tiensuu, Christopher Andersson, Patrik Rydén, Jörgen Johansson

**Affiliations:** 1 Department of Molecular Biology, Umeå University90187, Umeå, Sweden; 2 Laboratory for Molecular Infection Medicine Sweden (MIMS), Umeå University90187, Umeå, Sweden; 3 Umeå Centre of Microbial Research, UCMR, Umeå University90187, Umeå, Sweden; 4 Department of Mathematics and Mathematical Statistics, Umeå University90187, Umeå, Sweden; 5 Computational Life science Cluster (CLiC), Umeå University90187, Umeå, Sweden

## Abstract

Recently, several light receptors have been identified in non-phototrophic bacteria, but their physiological roles still remain rather elusive. Here we show that colonies of the saprophytic bacterium *Listeria monocytogenes* undergo synchronized multicellular behaviour on agar plates, in response to oscillating light/dark conditions, giving rise to alternating ring formation (opaque and translucent rings). On agar plates, bacteria from opaque rings survive increased levels of reactive oxygen species (ROS), as well as repeated cycles of light and dark, better than bacteria from translucent rings. The ring formation is strictly dependent on a blue-light receptor, Lmo0799, acting through the stress-sigma factor, σ^B^. A transposon screening identified 48 mutants unable to form rings at alternating light conditions, with several of them showing a decreased σ^B^ activity/level. However, some of the tested mutants displayed a varied σ^B^ activity depending on which of the two stress conditions tested (light or H_2_O_2_ exposure). Intriguingly, the transcriptional regulator PrfA and the virulence factor ActA were shown to be required for ring formation by a mechanism involving activation of σ^B^. All in all, this suggests a distinct pathway for Lmo0799 that converge into a common signalling pathway for σ^B^ activation. Our results show that night and day cycles co-ordinate a reversible differentiation of a *L. monocytogenes* colony at room temperature, by a process synchronized by a blue-light receptor and σ^B^.

## Introduction

Bacteria are found in almost all places in the environment, where they successfully occupy different niches. In order to do so, they have to be able to sense both chemical gradients and physical parameters, such as carbon availability, temperature or light. Sensing such signals might trigger multicellular behaviour in some bacterial species. One example is swarming where the bacteria move in a co-ordinated manner on a surface. In *Proteus mirabilis,* this event is induced by the inhibition of flagellar rotation (Morgenstein *et al*., 2010). Nutritional depletion initiates a complex pattern of multicellular behaviour in *Myxococcus xanthus*, including the formation of fruiting bodies, swarming and rippling, the latter allowing the bacteria to ‘hunt’ for a prey (Shimkets and Kaiser, 1982; Berleman *et al*., 2006; Berleman and Kirby, 2009). Other multicellular entities are biofilms that normally consist of several types of bacteria and are stabilized by an extracellular matrix (Lopez *et al*., 2010). Biofilms enhance bacterial survival during stress conditions like desiccation, presence of detergents and antimicrobials (Flemming and Wingender, 2010). The formation of biofilms as well as other multicellular activities is often triggered by a quorum sensing signal that co-ordinate the action of the entire population (Camilli and Bassler, 2006). *Listeria monocytogenes*, a Gram-positive bacterium can occasionally cause life-threatening infections (Hamon *et al*., 2006). In nature, *L. monocytogenes* can be found in soil, silage, sewage and vegetation and can among other things colonize different sprouts, which may act as vehicles for contaminating the food-chain (Gorski *et al*., 2004; Freitag *et al*., 2009). *Listeria* spp. are closely related to *Bacillus* species, but unlike the latter, *Listeria* spp. are unable to sporulate, which could prove disadvantageous when encountering soil-derived stress.

In this work, we show that colonies of *L. monocytogenes* display a synchronized colony behaviour by forming ordered circular rings (opaque and translucent rings) on an agar plate, in response to oscillating light and dark conditions at low temperatures (∼ 23°C). Bacteria in the opaque rings are more responsive to, and survive to a higher degree, oxidative stress than bacteria in translucent rings. Also, bacteria in opaque rings show an elevated long-term viability after repeated light and dark cycles compared with bacteria in translucent rings. We show that a blue-light receptor, acting through a σ^B^-dependent pathway, is required for ring formation. Several other factors required for ring formation were identified by transposon mutagenesis and many of them appear to control either the activity or the level of σ^B^. Interestingly, the well-characterized virulence factor ActA is required for σ^B^ activity at stress conditions. We also provide a mechanism showing that the formation of opaque rings requires light as well as nutrients.

## Results

### A synchronized colony differentiation in *L. monocytogenes* requires light/dark oscillations

**Figure 1:**
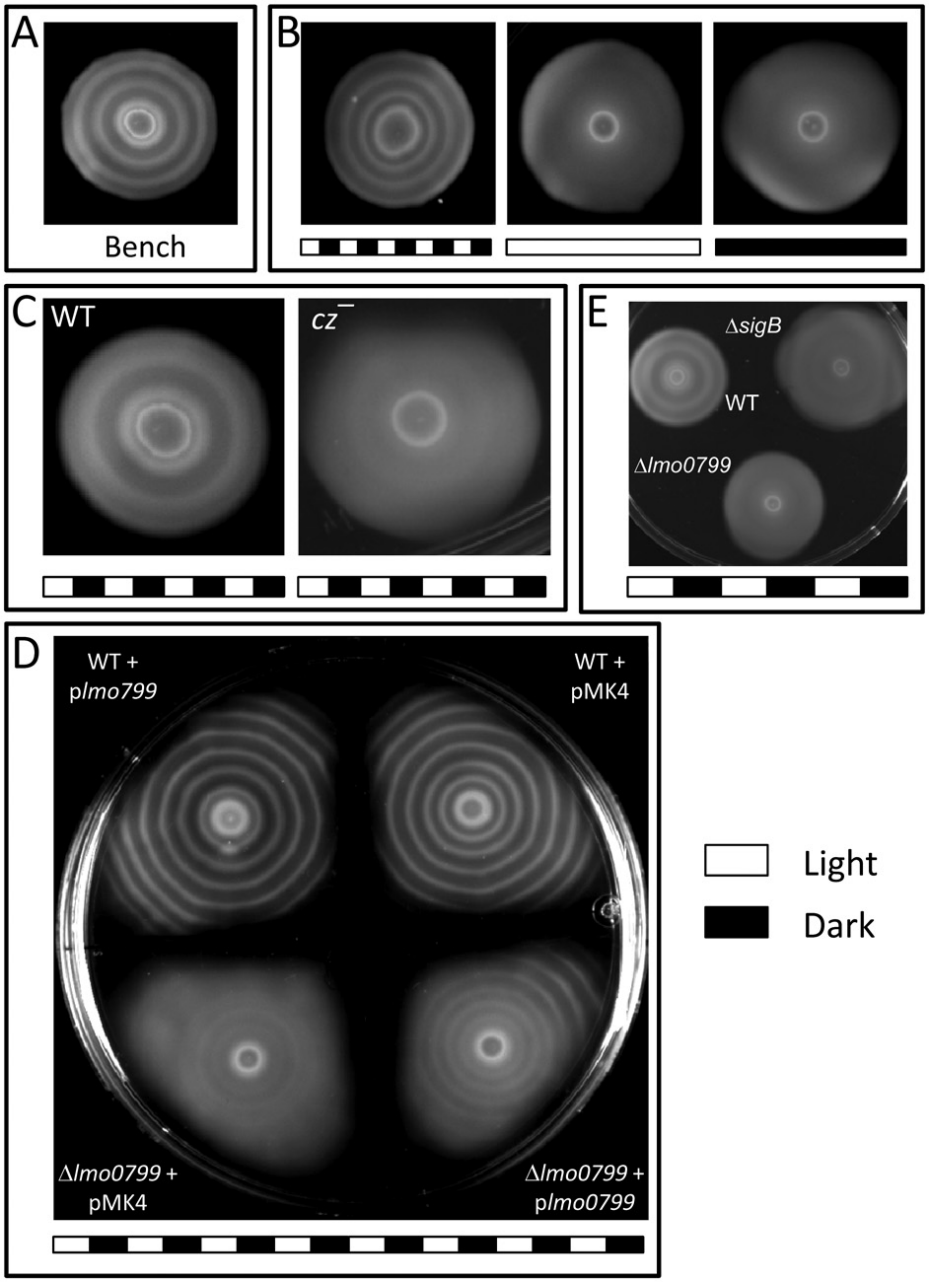
A blue-light receptor and oscillating cycles of light and dark control differentiation of a *Listeria monocytogenes* colony. A. Ring forming abilities on an agar plate on bench. Wild-type *L. monocytogenes* was inoculated on low-agar plates and incubated on the bench at room temperature for 96 h. B. Bacterial phenotypes on agar plates at different light and dark conditions. Wild-type *L. monocytogenes* was inoculated on low-agar plates and exposed to five cycles of 12 h light/12 h darkness (a total of 120 h, left panel), either to 120 h of constant light (middle panel) or to 120 h of constant darkness (right panel). An open bar indicates light conditions whereas a black bar indicates dark conditions. C. Bacterial phenotypes on agar plates with repetitive cycles of light and dark. Wild-type (WT) and *cz*− strains were inoculated on low-agar plates and exposed to four cycles of 12 h light/12 h darkness. D. Bacterial phenotypes on an agar plate with repetitive cycles of light and dark. Indicated strains were inoculated on a low-agar plate and exposed to eight cycles of 12 h light/12 h darkness. E. Ring-forming abilities of indicated strains on a low-agar plate exposed to four cycles of 12 h light/12 h darkness.

While analysing motility phenotypes in *L. monocytogenes*, one motility-agar plate with *Listeria* colonies was left on the bench at room temperature over a weekend. After 4 days, the *L. monocytogenes* colonies showed a distinct ring-shaped (bull's eye) form consisting of white (opaque) and dark (translucent) rings alternating from the middle and outward, with the entire colony being embedded within the agar (Fig. 1A). Similar looking ring-patterns have been designated consolidation zones (CZ) or terraces in *P. mirabilis* (Rauprich *et al*., 1996; Verstraeten *et al*., 2008). One possible explanation of the appearance of *Listeria* rings would be an ability to sense light. To test this, wild-type *L. monocytogenes* was plated and exposed to 12 h of light followed by 12 h of darkness, for five cycles (total of 120 h) or exposed to 120 h of constant light or 120 h of constant darkness. No rings could be observed for bacteria exposed to constant light or constant darkness (Fig. 1B). Instead, distinct rings were only observed when the bacteria were exposed to alternating light/dark conditions (Fig. 1B). By varying the time of light and dark exposure, rings of different width was obtained (Fig. S1). No rings were observed when *Listeria* were exposed to cycles of red light (625 nm) and darkness (Fig. S1).

### The formation of consolidation zones is dependent on a blue-light receptor

Concomitantly, when analysing phenotypes associated with a lysine riboswitch (located between *lmo0798* and *lmo0799*) (Toledo-Arana *et al*., 2009), a mutant (*cz−*) was isolated, unable to form rings during regular light/dark switching (Fig. 1C). Due to its phenotype, the *cz−* strain was hypothesized to harbour a mutation in *lmo0799*, encoding a putative blue-light receptor being homologous to the YtvA protein of *Bacillus subtilis*. Sequencing of *lmo0799* revealed a base deletion in the mutant strain, leading to a premature stop-codon. The YtvA protein harbours two domains; one LOV domain (light oxygen voltage) which binds flavin mononucleotide (FMN) non-covalently at dark conditions but where light triggers the formation of a covalent bond between a cysteine and FMN leading to signal transduction (Crosson *et al*., 2003; Losi *et al*., 2003). The other is a sulphate transporter, anti-sigma factor antagonist (STAS) domain, which has been suggested to be part of a high-molecular-weight, signal integration hub known as the stressosome, in *B. subtilis* (Aravind and Koonin, 2000; Akbar *et al*., 2001; Avila-Perez *et al*., 2006; Gaidenko *et al*., 2006). However, the stressosome complex has not yet been proven to exist in *L. monocytogenes*. The truncated Lmo0799 protein in the *cz−* strain had an intact LOV domain but lacked the STAS domain (Fig. S2). The above results suggest that the mutated form of Lmo0799 was responsible for the *cz−* phenotype. To verify this, a clean Δ*lmo0799* knockout strain was constructed (*Supporting information*). As predicted, absence of Lmo0799 prevented ring formation (Fig. 1D). Furthermore, when the Δ*lmo0799* strain was complemented with p*lmo0799* (i.e. the medium-copy-number, replicative plasmid pMK4 carrying the DNA fragment encoding the *lmo0799* gene), the bacterial ability to form rings on a low-agar plate was to a large degree re-established (Fig. 1D).

### The blue-light receptor affects global gene expression by acting through the stress sigma factor, σ^B^

Previously, it has been shown that the Lmo0799 homologue in *B. subtilis*, YtvA, require the STAS domain for σ^B^ activation at light conditions (Avila-Perez *et al*., 2009). A similar function has been pointed out for Lmo0799 in *Listeria* (Ondrusch and Kreft, 2011). We observed that the ring formation was dependent on σ^B^ (Fig. 1E). Also, expression of the genuinely σ^B^-regulated genes, *lmo0596* and *lmo2230* was highly induced at light conditions as compared with dark conditions (Fig. S3). Absence of Lmo0799 at light conditions decreased *lmo0596* and *lmo2230* expression to a level observed in the wild-type strains at dark, suggesting that light induction requires a functional blue-light receptor (Fig. S3A). This finding prompted us to examine if other σ^B^-regulated genes were also controlled in a similar manner. In total, we tested 14 additional σ^B^-regulated genes subdivided into different functional classes as defined previously (Hain *et al*., 2008; Toledo-Arana *et al*., 2009). Many σ^B^-regulated genes were induced by light exposure, in a blue-light receptor-dependent manner, with several of them being stress-responsive (Fig. S3A). Among these genes was *hfq*, encoding the RNA chaperone Hfq. Although we observed a light-induced expression of the Hfq protein, the absence of Hfq did not affect ring formation (Fig. S3B and C). A similar regulation of various σ^B^-regulated genes by the *L. monocytogenes* light receptor has previously been shown by another group (Ondrusch and Kreft, 2011).

### Lmo0799 decreases motility at light but not dark conditions by activating an antisense RNA

**Figure 2:**
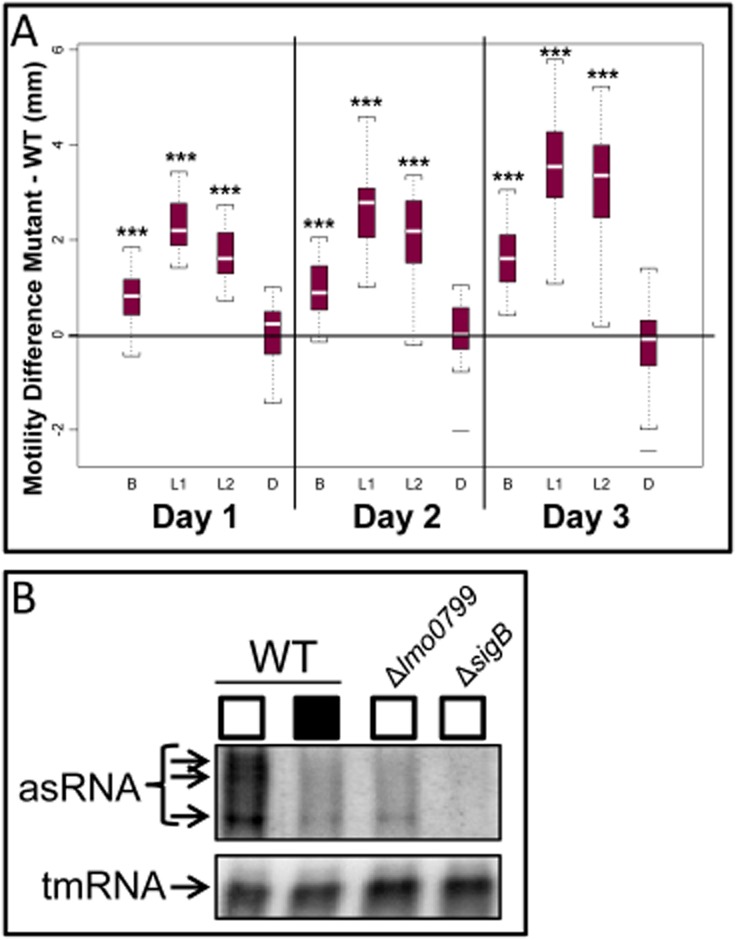
Light decrease *Listeria* motility in a mechanism requiring Lmo0799 and an antisense RNA. A. Wild-type (WT) and Δ*lmo0799* strains were inoculated on low-agar plates and incubated for indicated days on the bench (B); under blue-light-enhanced aquarium light (L1); under laboratory light (L2) or at darkness (D). Bacterial motility was scored daily and the difference between the Δ*lmo0799* mutant and the WT was plotted. The significant differences are denoted by stars: ‘***’ denotes Bonferroni corrected *P*-values of < 0.001. B. Northern blot analysis of asRNA expression at light and darkness. Indicated strains were grown at darkness (WT) or at light (WT; Δ*lmo0799* and Δ*sigB*) before RNA extraction and Northern blot. The membrane was hybridized with an asRNA RNA probe and a tmRNA (control) DNA probe respectively. Multiple bands of the antisense RNA were detected and the most prominent bands are highlighted by arrows.

Usually, the opaque rings are more narrow compared with translucent rings. To test if this was a result of reduced motility of *Listeria* at light conditions and if the light receptor was involved, motility tests were conducted. Bacteria lacking Lmo0799 were more motile compared with the wild-type bacteria at light conditions, but not at dark conditions (Fig. 2A). This suggested that motility of *Listeria* at light conditions was restrained by a mechanism requiring the light receptor. When exposed to light, a Δ*lmo0799* knockout strain overexpressing Lmo0799 on a plasmid (p*lmo0799*) was less motile compared with the WT strain harbouring the vector (Fig. S4). Recently, it was shown that σ^B^ activates expression of an antisense RNA (asRNA) lying on the opposite strand of listerial motility genes, thereby reducing motility at σ^B^ activating conditions (Toledo-Arana *et al*., 2009). RNA was extracted from different strains to investigate if light affected asRNA expression. Indeed, an increased expression of the antisense RNA was observed at light conditions, in a Lmo0799/σ^B^-dependent manner (Fig. 2B). In conclusion, light-triggered Lmo0799 activates σ^B^, which in turn induces expression of an antisense RNA previously shown to negatively affect motility (Fig. 2; Toledo-Arana *et al*., 2009).

### Opaque rings contain bacteria producing more extracellular polymeric substances (EPS)

**Figure 3:**
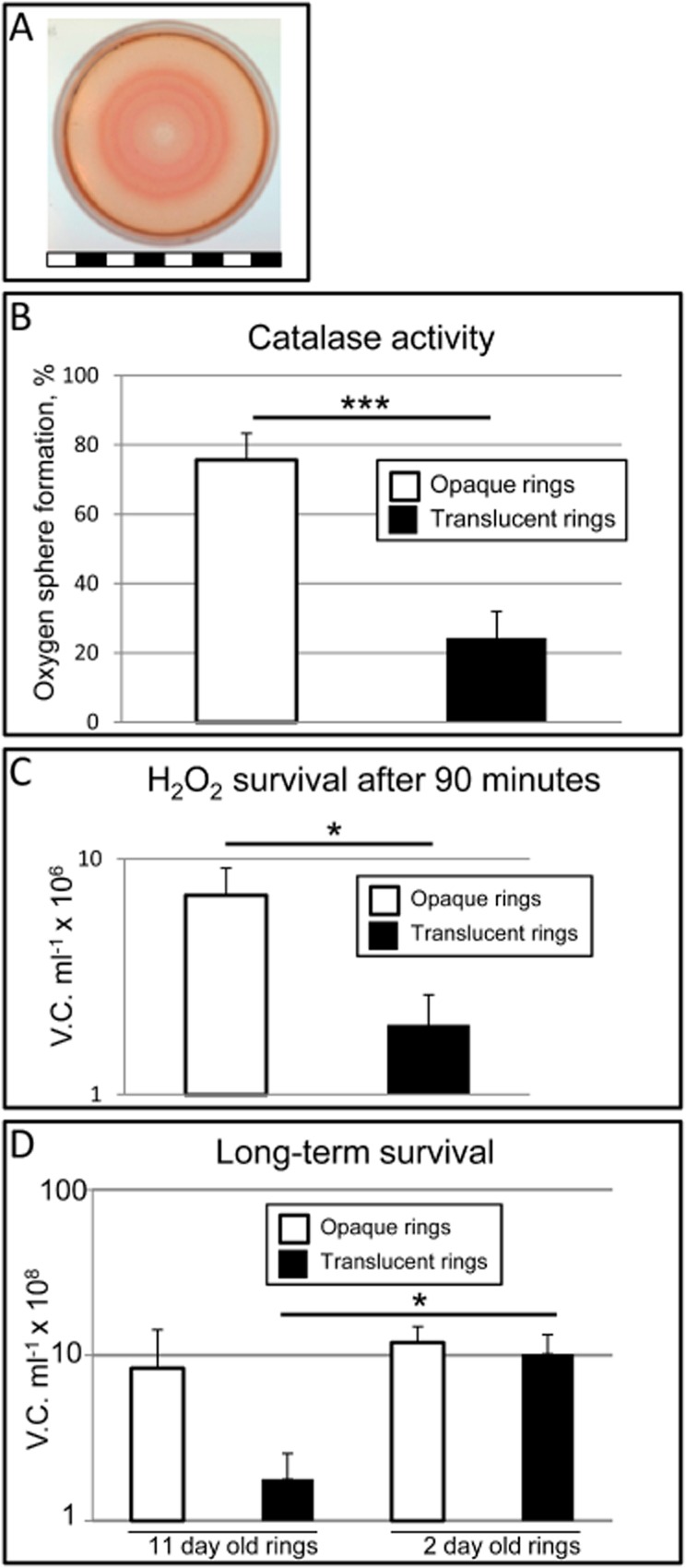
Opaque rings produce extracellular polymeric substances required for stress and long-term survival. A. A wild-type (WT) strain was inoculated on a low-agar plate containing 25 μg ml^−1^ Congo red and exposed to four cycles of 12 h light/12 h darkness. Red regions correspond to opaque rings, white regions to translucent rings respectively. B. Wild-type *L. monocytogenes* was inoculated on a low-agar plate and exposed to eight cycles of 12 h light/12 h darkness before addition of 1 M of H_2_O_2_ on a cross-section of the plate. The frequency of oxygen sphere formation at opaque and translucent rings was counted for 1 min and plotted as a fraction of 100%. *n* = 17 [*P* < 0.001 ***, Student's *T*-test (two-tailed)]. C. A wild-type strain was inoculated on a low-agar plate and exposed to five cycles of 12 h light/12 h darkness before bacteria were excised from 48-h-old opaque and translucent rings. Bacteria were resuspended in 1 ml of PBS and exposed to 60 mM of H_2_O_2_ for 90 min before plating. *n* = 3 [*P* < 0.05 *, Student's *T*-test (two-tailed)]. D. A wild-type strain was inoculated on a low-agar plate and exposed to 13 cycles of 12 h light/12 h darkness before bacteria from 2- or 11-day-old opaque and translucent rings were excised and plated. *n* = 3 [*P* < 0.05 *, Student's *T*-test (two-tailed)].

In *P. mirabilis*, the translucent rings are less dense, consisting of highly elongated, multinucleated bacteria, whereas the opaque rings harbour normal shaped bacteria (Morgenstein *et al*., 2010). In contrast to *P. mirabilis*, neither the size nor the numbers of *L. monocytogenes* bacteria (WT, Δ*lmo0799* and Δ*sigB* strains) differed between newly formed (∼ 2-day-old) opaque and translucent rings (Fig. S5A). Also, the number of dead and alive bacteria was similar in opaque and translucent rings as well as Δ*lmo0799* and Δ*sigB* strains (Fig. S5B). This suggests that the appearance of the opaque rings were due to structures on the surface of the bacteria and/or molecules produced and secreted by the bacteria in these zones. Transmission electron microscopy (TEM) on colonies from plates exposed to light and dark, indicated that bacteria isolated from opaque rings had a somewhat thicker cell wall as compared with bacteria from translucent rings and bacteria from the *lmo0799* mutant (Fig. S6). Bacteria from *Vibrio parahaemolyticus* colonies that display an opaque-like morphology, secrete more extracellular polymeric substances (EPS) compared with translucent colonies (Enos-Berlage and McCarter, 13). EPS is vaguely defined, and its content varies depending on growth conditions, as observed in *B. subtilis* (Marvasi *et al*., 2010). A previously used method to examine the presence and production of EPS is to use agar plates containing Congo red. EPS adsorbs Congo red, staining the colony red (Solano *et al*., 2002; Friedman and Kolter, 2004). Staining of *L. monocytogenes* motility plates by Congo red indicated the presence of EPS in opaque rings but not in translucent rings (Fig. 3A) Using ruthenium red, another EPS-staining dye, revealed similar results as for Congo red (data not shown). Taken together, our results suggest that the number of bacteria in translucent and opaque rings are similar, but that bacteria from opaque rings produce a higher amount of EPS.

### Bacteria from opaque rings survive stress better than bacteria from translucent rings, by a mechanism requiring Lmo0799

Are there any physiological differences between cells that comprise opaque versus translucent rings? Light-dependent processes are a major source of reactive oxygen species (ROS) in photosynthetic bacteria. However, also non-photosynthetic bacteria might encounter light-triggered ROS (Ziegelhoffer and Donohue, 2009). The LOV domain of certain blue-light receptors has been suggested to sense the oxidative state of the bacteria and fungi (Lamb *et al*., 2009; Purcell *et al*., 2010). In addition, functional σ^B^ contributes to *L. monocytogenes* survival at elevated ROS levels (Ferreira *et al*., 2001). It could therefore be speculated that Lmo0799 sense the amount of ROS and to protect the bacteria, activate σ^B^ through the stressosome. Alternatively, the expression of *lmo0799* could be induced by the presence of ROS, as been shown for *ytvA* in *B. subtilis* (Nakano *et al*., 2003). To test this latter hypothesis, we examined if ROS affected expression of *lmo0799* in order to modulate the number of active σ^B^ molecules. Presence of H_2_O_2_, but not the detergent stress inducer Triton X-100, increased expression of *lmo0799* at 37°C (Fig. S7). Absence of σ^B^ did not attenuate H_2_O_2_-induced expression of *lmo0799,* suggesting that the H_2_O_2_ effect on *lmo0799* expression was σ^B^-independent (Fig. S7). The increased expression of *lmo0799* in the presence of H_2_O_2_ indicated that the blue-light receptor could be involved in the bacterial response to ROS. Consequently, we tested if bacteria from opaque or translucent rings showed a differential catalase activity (scored as production of oxygen spheres, *Experimental procedures*) when exposed to 1 M H_2_O_2_. Bacteria from opaque rings showed a threefold higher ability of forming spheres compared with bacteria from translucent rings in presence of H_2_O_2_ (Fig. 3B, Supplemental Movie 1). Are bacteria from opaque rings more resistant against ROS than bacteria from translucent rings? To test this, bacteria from opaque and translucent rings were excised from the plate and exposed to 60 mM H_2_O_2_. After 90 min of H_2_O_2_ exposure, the number of bacteria from opaque rings was approximately fourfold higher than bacteria from translucent rings (Fig. 3C). Moreover, bacteria from colonies lacking Lmo0799 or σ^B^ showed a slightly decreased survival at 60 mM H_2_O_2_ as compared with bacteria from opaque colonies of the wild-type (Fig. S8). Since light-dependent processes could increase the level of ROS, the above data implicate that prolonged exposure to light would be more harmful for bacteria from translucent rings compared with opaque rings. The number of bacteria in opaque and translucent rings from 2- or 11-day-old rings was counted. The results show that the viability of bacteria was similar between opaque and translucent rings after 2 days, as seen before (Figs S5 and 3D). However, in 11-day-old rings, the number of bacteria in the translucent rings had dropped approximately fivefold compared with bacteria in 2-day-old rings, whereas the viability of bacteria in the opaque rings remained similar between 2- and 11-day-old rings (Fig. 3D). Our results therefore suggest that bacteria in the opaque rings are more resistant against ROS-mediated stress conditions and more adapted to long-term survival compared with bacteria from translucent rings in a mechanism dependent on Lmo0799 and σ^B^.

### Non-ring-forming transposon mutants are unable to activate σ^B^ at elevated stress conditions

In order to get a clearer picture of the cellular mechanism responsible for ring formation, a mariner transposon mutagenesis library of approximately 8000 colonies was created. Among these colonies, 48 were unable to form rings in response to light and dark oscillations (Table S1). The accuracy of the mutant bank was exemplified by transposon insertions in *sigB* and *lmo0799* yielding non-ring-forming colonies. Except the genes encoding unknown proteins, three main categories of genes were identified to be inactivated by the transposon: (i) genes encoding proteins involved in activation/repression of σ^B^ activity or affecting the integrity of the *sigB* transcript (*n* = 16). In *B. subtilis*, the activity of σ^B^ is increased during stress (such as elevated ROS) through inactivation of the anti-sigma factor RsbW by the anti-anti-sigma factor RsbV (Zuber, 2009). The Lmo0799 homologue YtvA has been suggested to be part of a protein complex known as the stressosome, important for activation/deactivation of σ^B^ in response to altered environmental conditions (Hecker *et al*., 2007). (ii) Genes encoding transport proteins and proteins involved in cell wall maintenance were found (*n* = 8). This class of mutants was particularly interesting, considering the putative difference in cell wall thickness and production of EPS identified between bacteria retrieved from light or dark conditions. (iii) Genes encoding regulatory proteins (*n* = 6). Among these were UvrA and Mfd, which form a complex able to release RNA-polymerases stalled at incorrect bases, commonly caused by ROS (Selby and Sancar, 2007).

**Figure 4:**
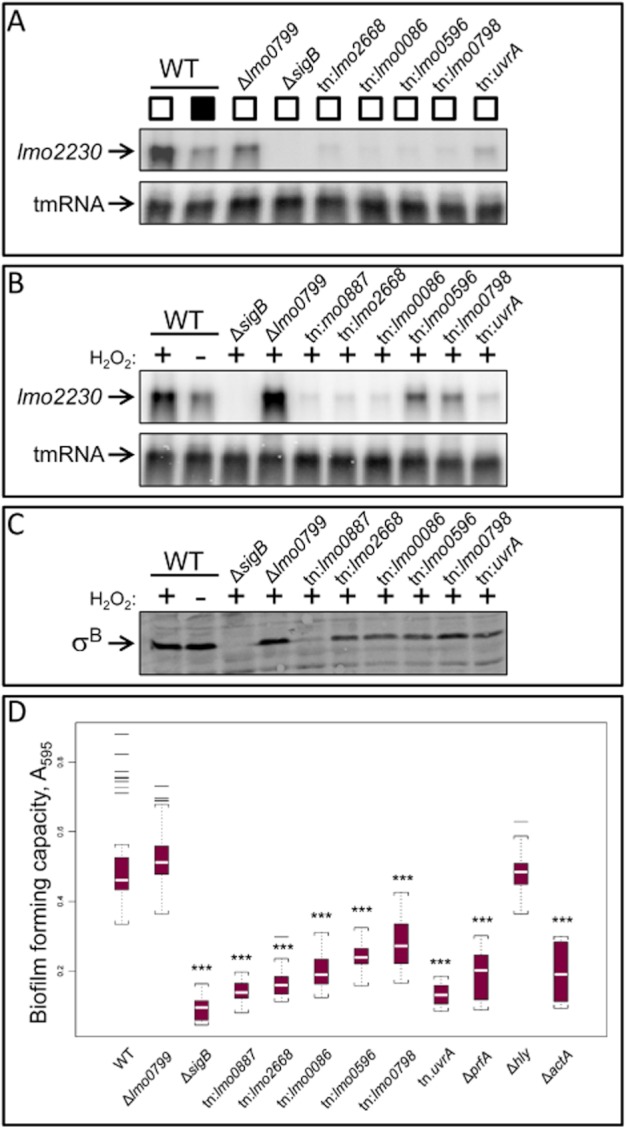
Transposon mutants unable to form rings are deficient for light and ROS induced σ^B^ activation as well as biofilm formation. A. Northern blot analysis of *lmo2230* expression. Indicated strains were grown at light or dark conditions before RNA extraction and Northern blot. The membrane was hybridized with *lmo2230* and tmRNA (control) specific DNA probes. B. Northern blot analysis of *lmo2230* expression. Indicated strains were grown in darkness in presence (+) or absence (−) of 60 mM H_2_O_2_ before RNA extraction and Northern blot. The membrane was hybridized with *lmo2230* and tmRNA (control) specific DNA probes. C. Western blot analysis of σ^B^ expression. Indicated strains were grown in darkness in presence (+) or absence (−) of 60 mM H_2_O_2_ before protein extraction and Western blot. The membrane was hybridized with an α-σ^B^ specific antibody. D. Indicated strains were inoculated in microtitre plates at light condition for 48 h before staining with crystal violet and *A*_595_ measurement. The normalized signals of each strain were plotted and compared with the wild-type (WT) strain and the significant differences (Bonferroni corrected *P*-values < 0.001) were denoted with ‘***’.

The mutants identified in category 1 suggested that this transposon mutant class was connected to the function of σ^B^. Since the ring formation was dependent on cycles of light and dark, we were interested to examine whether light-induced σ^B^ activation was affected in the transposon mutants of categories 2 and 3. To test this, a subset of transposon mutants was grown at light-exposing conditions (Fig. 4A). As manifested by the expression of the genuinely σ^B^-dependent gene *lmo2230,* light-induced σ^B^ activation was almost abolished in all but one transposon mutant [located in *uvrA –* encoding a DNA repair protein (Truglio *et al*., 2006)] which displayed an intermediate σ^B^ activity. The connection between light and ROS production as well as the decreased survival of Δ*lmo0799* and Δ*sigB* strains at elevated ROS levels prompted us to examine whether H_2_O_2_-induced σ^B^ activation was affected in the above transposon mutants. ROS-induced σ^B^ activation was almost abolished in all but two transposon mutants, compared with the wild-type (Fig. 4B). In those two transposon mutants, the σ^B^ activity was slightly attenuated compared with wild-type activity, and the transposons were located in a lysine riboswitch (abolishing expression of the gene-encoding lysine permease Lmo0798) and in the promoter region of the gene encoding the membrane protein Lmo0596 respectively (Table S1). Interestingly, the level of the σ^B^ protein was only slightly decreased (a maximum of threefold) in the above tested transposon mutants apart from one. Previously, expression of σ^B^-dependent genes in *L. monocytogenes* have been shown to mainly depend on the activity of σ^B^ (O'Byrne and Karatzas, 2008) indicating that the proteins encoded by the transposon mutants act on the level of σ^B^ activity (Fig. 4C) although a minor effect might be exerted at the σ^B^ protein level. The only exception was the transposon mutant disrupting *lmo0887* expression, probably due to a polar effect of the transposon on *sigB* expression since they are part of the same transcript (Toledo-Arana *et al*., 2009). To verify if the decreased *lmo2230* expression in the transposon mutants indeed was due to the transposon insertion, a complementation approach was undertaken. By expressing *lmo0596*, *lmo0798* and *lmo0799 in trans* in the respective transposon mutants, we were able to completely or partially restore expression in *lmo2230* (Fig. S9). Expression of *lmo2230* could not be restored in transposon mutants expressing *lmo2668* and *uvrA in trans*, probably since these genes are part of larger operons giving an effect of the transposon mutant through another gene in the operon. In addition, due to its size, we were not able to make a construct expressing *lmo0086*. Most importantly though, our data for *lmo0596*, *lmo0798* and *lmo0799* show that the effect of *lmo2230* expression is indeed mediated by transposon insertions in specific genes and not due to indirect effects of the transposon itself. Therefore, the function of these gene products were studied in more detail for the remainder of the article. In conclusion, our results suggest that light as well as ROS can induce σ^B^ activation through a common pathway.

Since both σ^B^ in *L. monocytogenes* and ROS in other bacteria have been shown to influence biofilm formation (van der Veen and Abee, 2010; Donati *et al*., 2011), the transposon mutants and the Δ*sigB* and Δ*lmo0799* strains were tested for their abilities to form biofilm compared with the wild-type strain. All transposon mutant strains tested as well as the Δ*sigB* strain showed a reduced biofilm formation (Fig. 4D). The reduced biofilm formation in the Tn:*lmo0887* strain is probably due to reduced expression of *sigB* and this strain was not considered further. The biofilm formation also displayed a light/dark difference; with biofilms being more pronounced at light conditions compared with dark conditions (data not shown). Unexpectedly, the Δ*lmo0799* strain formed biofilms as prominent as the wild-type, indicating that biofilm formation is independent of Lmo0799 and that none of the tested mutants exerted their negative effect on biofilm formation by affecting Lmo0799 activity.

### l-lysine inhibits ring formation

One of the transposon mutants was located in a lysine riboswitch, eliminating expression of the downstream gene (*lmo0798*) encoding a lysine permease (Table S1). At high levels of lysine, the lysine riboswitch forms a terminator structure, abolishing transcription of *lmo0798* (Toledo-Arana *et al*., 2009). In contrast, absence of lysine forces the formation of an anti-terminator structure, allowing expression of *lmo0798*. We were therefore interested to examine whether lysine could affect ring formation. Presence of l-lysine was able to inhibit ring formation on plate (Fig. S10). As shown in Fig. 4A–C, Lmo0798 was important for σ^B^ activation at light exposure, but less important at elevated ROS levels. Despite this was *lmo0798* expression not stimulated by light exposure (data not shown). In conclusion, l-lysine inhibits ring formation, presumably by decreasing *lmo0798* expression, which in turn is required for σ^B^ activation at light conditions.

### The virulence factor ActA is required for ring and biofilm formation, but also controls σ^B^ activity

**Figure 5:**
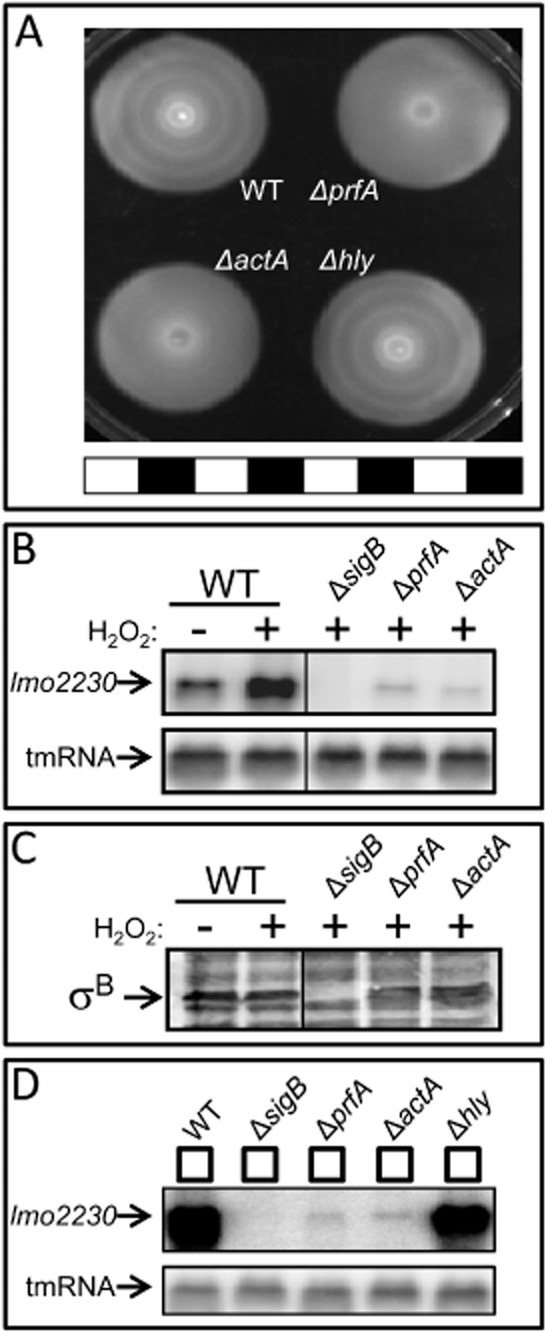
ActA is required for ring formation and controls σ^B^ activity. A. Indicated strains were inoculated on a low-agar plate and exposed to four cycles of 12 h light/12 h darkness. B. Northern blot analysis of *lmo2230* expression. Indicated strains were grown in darkness in presence (+) or absence (−) of 60 mM H_2_O_2_ before RNA extraction and Northern blot. The membrane was hybridized with *lmo2230* and tmRNA (control) specific DNA probes. C. Western blot analysis of σ^B^ expression. Indicated strains were grown in darkness in presence (+) or absence (−) of 60 mM H_2_O_2_ before protein extraction and Western blot. The membrane was hybridized with an α-σ^B^ specific antibody. D. Northern blot analysis of *lmo2230* expression. Indicated strains were grown at light conditions before RNA extraction and Northern blot. The membrane was hybridized with *lmo2230* and tmRNA (control) specific DNA probes.

It has recently been shown that *L. monocytogenes* lacking the virulence regulator PrfA form reduced biofilm as compared with a wild-type strain (Lemon *et al*., 2010). In light of our findings correlating ring formation with biofilm development, we were interested to examine if a Δ*prfA* strain could form rings on plates exposed to cycles of light and dark. A Δ*prfA* strain was not able to form rings on an agar plate in response to cycles of light and dark or biofilm (Figs 4D and 5A). In line with this, a *Listeria innocua* strain (a non-virulent *Listeria* strain lacking ActA) displayed a weaker ring-formation capacity compared with *L. monocytogenes* (Fig. S11). Two genes regulated by PrfA whose gene products potentially could be involved in ring- and biofilm formation are Listeriolysin O (LLO) and ActA (encoded by *hly* and *actA* respectively). These proteins are very important during the intracellular growth of *Listeria* inside eukaryotic cells, with LLO enabling the bacterium to escape from phagosomes and ActA mediating intracellular movement by recruiting an actin polymerization complex (Hamon *et al*., 2006). When tested on agar plates under oscillating light and dark conditions, the Δ*hly* strain formed rings as prominent as the wild-type (Fig. 5A). The Δ*hly* strain also formed biofilm equally well as the wild-type (Fig. 4D). In contrast, the Δ*actA* strain was not able to form rings on agar plates in response to cycles of light and dark (Fig. 5A). Absence of ActA also reduced the capacity of the bacteria to form biofilms explaining the inability of *prfA* mutants to form biofilms or rings, although other PrfA-regulated genes might also participate in the ring formation (Fig. 4D) (Lemon *et al*., 2010). During infection at 37°C, ActA expression is subject to a very tight regulation and is massively induced intracellularly in a process requiring PrfA (Freitag *et al*., 2009). Western blot experiments revealed that both PrfA and σ^B^ was required for full ActA expression at 23°C (Fig. S12). Intriguingly, σ^B^ is not important for *actA* expression at 37°C and when *Listeria* is exposed to blood (Toledo Arana *et al*., 2009). Is ActA necessary for σ^B^ activity? To examine this, the amount of *lmo2230* expression was monitored in strains lacking ActA or PrfA. The results show that σ^B^ activity was attenuated (reduced *lmo2230* expression) in the absence of ActA or PrfA, in a manner not affecting the amount of σ^B^ protein at elevated ROS levels (Fig. 5B and C). Also, *lmo2230* expression was almost abolished in Δ*prfA* and Δ*actA* strains but not in a Δ*hly* strain when exposed to light (Fig. 5D). Importantly, expressing *actA in trans* on a plasmid partially restored *lmo2230* expression, showing that the observed effect indeed is mediated through the absence of ActA (Fig. S9). In conclusion, our data indicate that ActA and σ^B^ are part of a regulatory loop controlling the level/activity of each other.

### Opaque rings are only formed when bacteria are metabolically active

**Figure 6:**
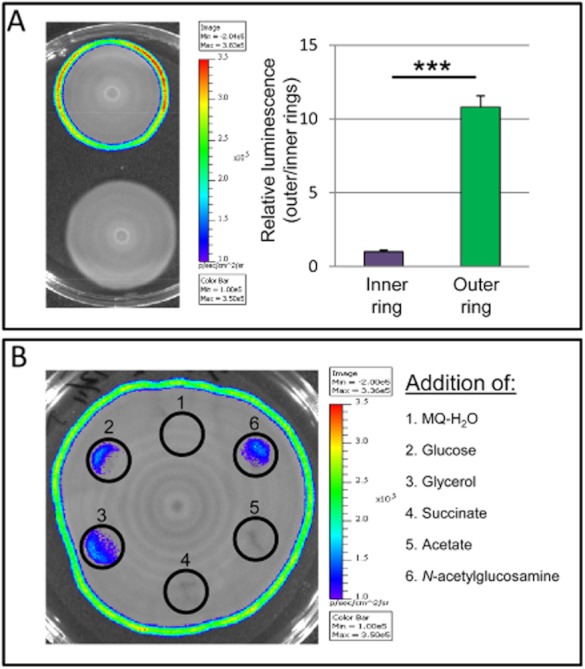
Bacteria in inner rings enter a resting state due to carbon deficiency. A. Wild-type (WT) *L. monocytogenes* strains without (lower colony) or with (upper colony) a chromosomal fusion (pPl2luxP_help_) were inoculated on low-agar plates and exposed to five cycles of 12 h light/12 h darkness. Luciferase expression was measured at 12- and 60-h-old rings from the luciferase expressing strain and plotted as relative light expression [*P* < 0.001 ***, Student's *T*-test (two-tailed)]. B. The strain harbouring the pPl2luxP_help_ fusion was inoculated on low-agar plates and exposed to cycles of 12 h light/12 h darkness. One micromole of different carbon sources (glucose; glycerol; *N*-acetylglucosamine: succinate or acetate) were spotted onto the agar plate (black circles) and luciferase expression was measured after 30 min.

How are the translucent rings prevented to form opaque rings when exposed to light? Since the amount of bacteria did not vary between newly formed opaque and translucent rings (Figs S5 and 3D), we were interested to examine if the bacteria showed a different metabolic activity throughout the rings. An indirect way of measuring the intracellular energy is to measure luciferase expression, which requires ATP for activity. For this, a constitutively active and chromosomally integrative reporter plasmid (pPL2luxP_help_) was used (Riedel *et al*., 2007). Strikingly, prominent luciferase expression was only observed in the newly formed outermost rings, being ∼ 10-fold higher than the inner rings formed less than 48 h ago (Fig. 6A). The above results indicate that bacteria in all rings, except the most newly made, enter a resting state and become metabolically inactive. To investigate if the resting status of the bacteria was due to nutrient limitation, 1 μmol of different carbon sources (glucose, glycerol, *N*-acetylglucosamine, succinate and acetate) were added on top of an agar plate containing wild-type *L. monocytogenes*. Addition of glucose, glycerol and *N*-acetylglucosamine, but not succinate or acetate, induced luciferase expression within 30 min (Fig. 6B). This is in line with previous results showing that succinate and acetate are poor carbon sources for *Listeria* growth (Premaratne *et al*., 1991). The carbon limitation in the inner rings thereby explains why translucent rings are not able to convert to opaque rings when later exposed to light.

**Figure 7:**
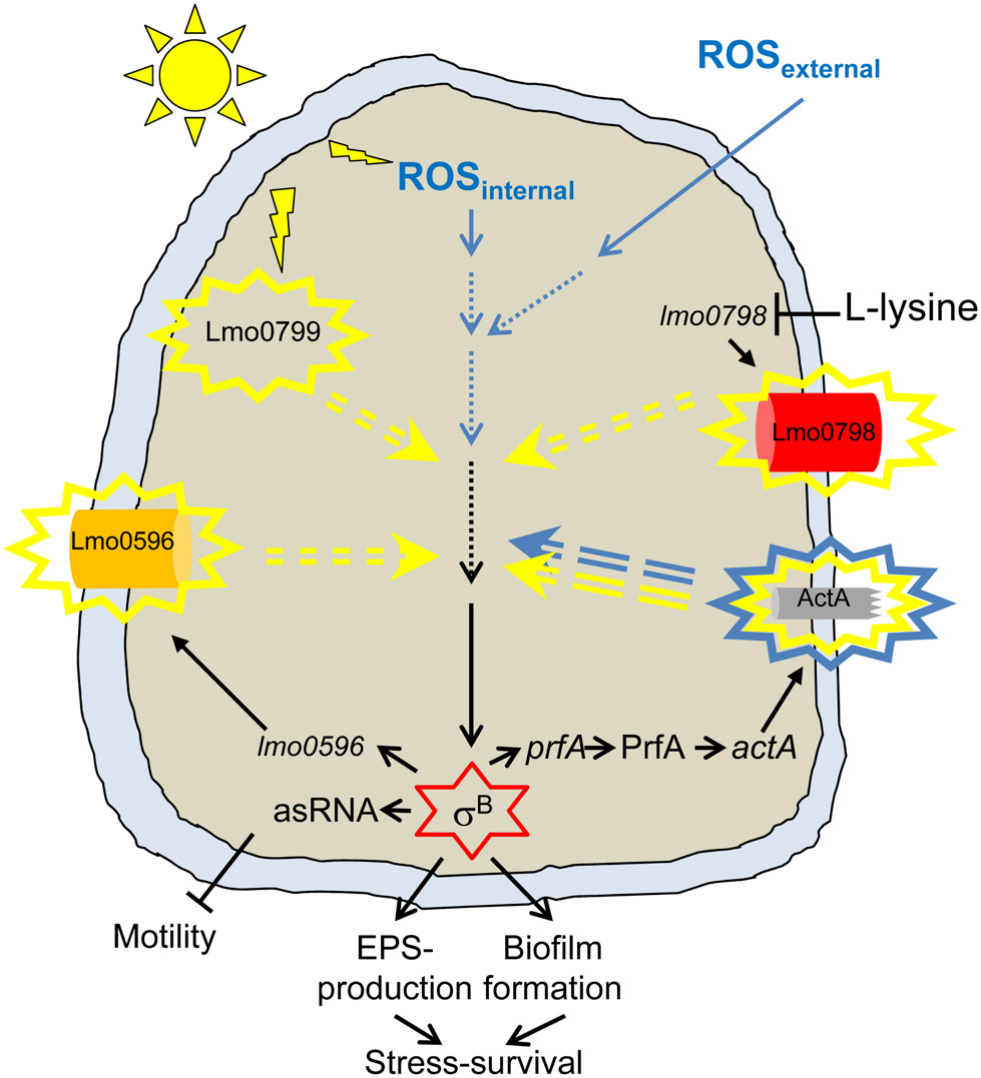
Schematic model. At light conditions (sun), Lmo0799 becomes activated, resulting in liberated σ^B^. The presence of reactive oxygen species (ROS – in blue) also leads to freed σ^B^. Free σ^B^ activate, among others, genes important for extracellular polymeric substances (EPS), an antisense RNA inhibiting bacterial motility, *prfA* and *lmo0596* expression respectively. PrfA in turn activates *actA* expression. *lmo0798* expression is blocked by l-lysine. ActA, Lmo0596 and Lmo0798 could function independently and later converge into a common σ^B^ activating pathway. Proteins being important for light-dependent σ^B^ activation are shown in yellow, proteins being important for ROS-dependent σ^B^ activation are shown in blue. Dashed lines indicates non-defined pathways.

Based on the above results, the following mechanism for ring production is suggested: if exposed to light, the Lmo0799/σ^B^ pathway allows the outermost bacteria to produce EPS, leading to the formation of a distinct opaque ring. In contrary, if the outermost bacteria are formed at dark conditions, the Lmo0799/σ^B^ pathway remains inactivated and a translucent ring is formed (low level of EPS) (Fig. 7).

## Discussion

In this work, we show that the Gram-positive soil bacterium *L. monocytogenes* is able to display a synchronized multicellular behaviour (forming opaque and translucent rings) in response to cycles of oscillating light and dark (Fig. 1) at low temperatures (23°C) on agar plates. Constant light or dark conditions abolish the formation of rings, suggesting a possible role for the phenomenon in nature when the bacteria faces cycles of night and day. What function do the formation of opaque and translucent rings play? Our results suggest that the number of bacteria in opaque and translucent rings initially do not differ (Figs S5A and 3D). However, bacteria in opaque rings survive repeated cycles of light and dark better than bacteria in translucent rings (Fig. 3D). Also, bacteria in opaque rings respond to and survive increased levels of ROS better than bacteria from translucent rings (Fig. 3B and C). The opaque rings contain bacteria producing a higher amount of EPS than bacteria from translucent rings (Fig. 3A). Current work in the laboratory focuses at revealing the molecular constituents of the EPS at light conditions. Our results suggest that light-triggered formation of opaque rings could mediate long-term survival of *L. monocytogenes* in the environment, such as soil. This mechanism is especially intriguing since *Listeria*, despite being closely related to spore-forming *Bacillus* species, is unable to sporulate.

Within this work, a blue-light receptor was identified to co-ordinate the ring formation (Fig. 1). The blue-light receptor exerts its regulation through the activity of the stress-sigma factor σ^B^, as has previously been shown (Avila-Perez *et al*., 2006; Ondrusch and Kreft, 2011). Several σ^B^-regulated genes were indeed shown to respond to light and dark in a blue-light receptor-dependent manner (Fig. S3A). Many of these genes are involved in stress response and functional Lmo0799 and σ^B^ supports survival of *L. monocytogenes* at elevated ROS levels (Fig. S8). Our results suggest that light-derived Lmo0799 activity redirect energy resources from motility (flagella-movement) to EPS production (Figs 2 and 3).

By a transposon mutagenesis screening, we identified 48 additional gene products required for ring formation. Most of the tested transposon mutants showed a reduced σ^B^ activity at both light and ROS stress (Fig. 4A and B). Interestingly however, some of the tested mutants displayed a varied σ^B^ activity depending on the stress condition. This is exemplified by an inactivated expression of *lmo0596* (encoding an unknown membrane protein) or *lmo0798* (encoding a lysine permease) leading to a more strongly attenuated σ^B^ activity at light conditions, compared with elevated ROS levels (Fig. 4A–C). However, *lmo2230* expression is nonetheless lower at increased ROS levels in the tn*:lmo0596* and the tn*:lmo0798* strains compared with the wild-type strain. The gene encoding the membrane protein Lmo0596 is especially interesting since it was observed to be controlled by light and dark through the light receptor and σ^B^ (Fig. S3A). A transposon mutant abolishing expression of *lmo0596* showed a reduced σ^B^ activity at light conditions and a diminished biofilm formation (Table S1, Fig. 4A–D). Hence, Lmo0596 is regulated by σ^B^, but also control σ^B^ activity in a regulatory loop (Fig. 7). A similar scenario is found for ActA which is required for ring and biofilm formation (Figs 4D and 5A). σ^B^ is required for maximal ActA expression, but ActA in turn controls σ^B^ activity (Figs 5B–C and 7). The feedback mechanism exerted by Lmo0596 and ActA on σ^B^ activity is intriguing and their possible role in σ^B^ activation requires further investigation. The lysine permease Lmo0798 represents another case; Absence of lysine permease expression (by either a transposon insertion or excess lysine) abolishes ring formation (Table S1 and Fig. S10), and also reduces σ^B^ activity (Fig. 4A). Whether Lmo0596, Lmo0798 and ActA act individually or together is unclear but it is interesting that these proteins and several of the transposon mutants unable to form rings encode membrane-spanning proteins (Table S1, Fig. 7). It should be noted that only one σ^B^-dependent gene tested (*lmo0596*) was also identified in the screen of transposon mutants unable to form rings in response to cycles of light and dark (Table S1). This could indicate that almost all genes regulated by σ^B^ act as effector proteins of ring formation and not as regulators of σ^B^ activity. It could also suggest a redundancy between σ^B^-regulated genes where they can substitute each other.

The amount of ROS that potentially could be very harmful for the cell is increased after exposure to light (Ziegelhoffer and Donohue, 2009). Our data suggest that *L. monocytogenes,* at least partially, handles an increase in ROS by enhancing the expression of *lmo0799* (Fig. S7), whose gene product activates several ROS-responsive genes through a σ^B^-dependent pathway. Absence of the light receptor Lmo0799 decreases the survival of *Listeria* at conditions of elevated ROS (Fig. S8). The light oxygen voltage (LOV) domain could play a role in sensing ROS, as it has been suggested to do in other bacteria and in fungi (Lamb *et al*., 2009; Purcell *et al*., 2010). Here, we observe an increased expression of *lmo0799* in the presence of ROS, allowing for an alternative integration mechanism of light and ROS sensing into the σ^B^ pathway. Interestingly, the Lmo0799 protein has recently been suggested to have a different photocycle activity as compared with YtvA due to the lack of a conserved arginine in the flavin binding site (Chan *et al*., 2012). However, as identified by the transposon mutant library, *Listeria* uses several other gene products acting through the σ^B^-dependent pathway (independent of Lmo0799) to respond to an increase in ROS levels. Absence of any of these gene products attenuates the increased σ^B^ activity during elevated H_2_O_2_ levels or light exposure. Clearly, σ^B^ is absolutely required to integrate signals from different stress conditions into one regulatory outcome and consequently, absence of σ^B^ makes the bacteria more sensitive to ROS and light (Figs 7 and S8). We believe that the light receptor Lmo0799 in *Listeria* functions as a first sensor interpreting light as a precursor of increased ROS, alerting the bacterium. Subsequently, when the level of ROS is elevated, the other ROS sensors become activated leading to an even stronger σ^B^ activation, thereby making Lmo0799 redundant. Lmo0799 as well as the other ROS sensors mediate their signal through activation of the σ^B^ pathway, which makes it possible that they act through the putative stressosome complex. Activated σ^B^ induces expression of several genes that ultimately will allow the bacteria to survive increased light/ROS levels. We suggest that the stressosome is able to recruit specific partners depending on stress conditions.

One important aspect of our findings is that even daylight can induce multiple stress responses in *L. monocytogenes* as has been shown previously with a modest increase in SigB activity in response to light (Ondrusch and Kreft, 2011). This is in contrast to *B. subtilis*, where the effect of the blue-light receptor YtvA can only be observed if another stress condition triggering σ^B^ activity is added simultaneously, or if YtvA is overexpressed (Gaidenko *et al*., 2006; Avila-Perez *et al*., 2009). We therefore recommend that future experiments with *L. monocytogenes* (and possibly also other bacteria) should be carried out at dark conditions, to avoid light-induced stress-effects. Also, most of the described effects herein are only observed at room temperature (23°C), indicating a role for the light-sensing mechanism at non-infectious temperatures.

Recently, it was shown that *Listeria* remain infectious also after several generations in stationary phase (Bruno and Freitag, 2011). It is however unclear how the virulence factors are kept on the chromosome if they do not offer a selective advantage for the bacterium at non-infectious conditions. Based on our results, it could be hypothesized that the role of PrfA and ActA at such conditions could give a selective advantage for maintaining the virulence factors.

The data in this article, together with results from other groups, show that non-phototropic bacteria harbour an elaborate mechanism to sense light and other stress signals and integrate them into a specific pathway.

## Experimental procedures

### Oligonucleotides and antibodies

Oligonucleotides used in this study are listed in Table S2. Strains, plasmids and antibodies used in this study are listed in Table S3.

### Bacterial culturing conditions

*Listeria monocytogenes* strains were grown in Brain Heart Infusion (BHI) (Fluka) at 23°C, unless otherwise stated, with aeration. Chloramphenicol and erythromycin was added at a concentration of 7 μg ml^−1^ where needed. For growth in light or dark conditions, overnight cultures were grown in dark (tubes wrapped in aluminium foil), diluted 100-fold in BHI and grown in light or dark conditions until the desired optical density. In light conditions, culture flasks were placed under an aquarium light enhanced for blue-light (Power Glo, 20W, T8) with an average light intensity at the flasks of 29.7 μmol m^−2^ s^−1^. In dark conditions, culture flasks were tightly wrapped in aluminium foil. For growth at stress conditions, overnight cultures were diluted 100-fold and grown until the desired optical density. Thereafter, 0.15% H_2_O_2_ or 2% Triton X-100 was added and cultures were grown an additional 10 min before samples were withdrawn for RNA isolation.

### RNA isolation

RNA isolation was performed as described (Loh *et al*., 2009) with some minor changes. Bacteria were grown to OD_600_ = 0.8. TRI-reagent Soln. (Ambion) was used to isolate RNA.

### Northern blot

Twenty micrograms of RNA was separated on a 1.2% agarose gel containing 1× HEPES buffer (10× HEPES buffer: 0.2 M HEPES 50 mM NaAc, 10 mM EDTA, adjusted to pH 7) and 7.3% formaldehyde. The gel was run in 1× HEPES buffer at 100 V for 4 h and the RNA was transferred to a Hybond–N membrane (Amersham) by capillary transfer in 20× buffer SSC. The membranes were cross-linked, pre-hybridized in Rapid hyb buffer (Amersham) for about 2 h at 60°C and then hybridized with ^32^P α-labelled DNA fragments using Megaprime DNA labelling system (Amersham) at 60°C overnight. DNA fragments were amplified with PCR using corresponding primers (Table S1). Membranes were washed (0.5% SDS, 2× SSC, room temperature for 15 min followed by 0.5% SDS, 0.1× SSC 60°C for 15 min), exposed in a phosphorimager cassette and developed using the STORM machine (Molecular Dynamics). For RNA probing, the probe was generated by performing *in vitro* transcription using T7 MAXIscript Kit In vitro transcription kit (Ambion) according to the manufacturer. The DNA template was generated by PCR using primers T7-lmo0676 fwd (containing the T7 promoter) and lmo0676 rev (Table S1).

### Motility assay

Two microlitres of overnight cultures were spotted on BHI plates containing 0.3% agar (low-agar, Oxoid). Colonies were allowed to grow at room temperature and plates were photographed using Chemidoc (Bio-Rad) with EpiWhite Illumination. For motility assays at dark conditions, plates were wrapped in aluminium foil. For motility assays at light conditions, plates were placed either under normal laboratory light or under an aquarium light enhanced for blue-light (Power Glo, 20W, T8) as indicated. At bench conditions, plates were exposed to regular light at the laboratory bench. For motility assays with different light/dark intervals, either plates were placed in a dark room under an aquarium light enhanced for blue-light (Power Glo, 20W, T8), equipped with a timer allowing the light to be turned on and off at indicated intervals, or plates were manually switched between dark and light conditions. The statistical analysis was performed as follows: the experiment included 10 plates each containing a wild-type and a mutant colony. Measurements were performed at the start of the experiment and at every 24 h for 3 days using a digital vernier-micrometer. The motility was quantified by measuring the diameter of the colonies. Two measure values for each colony were obtained by measuring the diameter at two places perpendicular to each other. The inoculum size was subtracted from the measuring values and for each colony the average of the two corrected measurements was calculated. For each plate and day the difference between the wild-type and mutant motility was calculated and the Wilcoxon signed rank test was used to test if there was a significant difference between strains. Bonferroni correction was used to address the problem of multiple comparisons.

### Lysine motility assay

Two microlitres of EGDe and Δ*lmo0799* overnight cultures were spotted on 0.3% agar (Oxoid) BHI plates containing 0 (negative control), 20, 30 and 40 mM l-lysine. Plates were grown at room temperature and exposed to four cycles of 24 h light/24 h dark. For light conditions plates were placed under normal laboratory light and for dark conditions plates were wrapped in aluminium foil. Plates were photographed using Chemidoc (Bio-Rad) with EpiWhite Illumination.

### Construction and complementation of deletion mutants and isolation of the *cz−* mutant

Precise deletions of *lmo0799*, *actA* and *hly* was generated using the pMAD suicide vector. Approximately 1000 bases upstream and downstream of *actA* and *hly*, respectively, was amplified and inserted into pMAD. The *actA* gene was deleted from the first codon (the first G in the start-codon GUG is maintained on the chromosome) until the A base just after the UAA stop-codon. Hence, the entire ActA protein was removed but nothing else from the chromosome. To achieve allelic exchange, we followed the protocol of Arnaud *et al*. (2004). The Δ*lmo0799* strain was complemented using the pMK4 plasmid with an insertion spanning approximately 130 bases upstream of the *lmo0799* start codon, to a region just downstream of the lysine riboswitch terminator. Detailed procedures is provided in *Supporting information* (Supporting Experimental Procedures). The *cz−* mutant was isolated when a deletion of a lysine riboswitch (lying downstream of *lmo0799*) was made. When examining the Δ*lysine* riboswitch strain, a phenotype not correlated with the lysine riboswitch was observed. By sequencing the flanking regions, a base-deletion in the gene *lmo0799* was observed, leading to a truncated Lmo0799 protein.

### Biofilm assays

Biofilm assays were performed as previously described (Harvey *et al*., 2007) with some modifications. Colonies were grown in 5 ml of TSB overnight at 37°C. Twenty microlitres from the overnight cultures were inoculated in 10 ml of TSB and grown at 26°C either at light or at dark conditions ∼ 16 h. Two hundred and fifty microlitres from these cultures were diluted into 5 ml of TSB and vortexed 30 s before 100 μl were transferred into wells of sterile microtitre plates (96 wells, non-tissue treated, U-shaped bottom, polystyrene plates, Falcon). Plates were incubated at room temperature either in light conditions (under aquarium light) or in dark conditions (plates wrapped in foil) for 48 h. After incubations, cultures were removed and wells were washed with sterile water, stained with 1% crystal violet and washed with sterile water as previously described. One hundred microlitres of ethanol (95%) was added to destain the biofilm and the concentration of biofilm staining was measured at an absorbance of *A*_595_ using Infinite 200 plate reader (Tecan). The statistical analysis was performed as follows: The experiment included 12 plates (six performed in light conditions) were each plate contained a number of strains each with eight biofilm measurements. The wild-type strain was represented on all 12 plates while the 13 mutant strains (see Fig. 4D) where represented on 4–12 plates. The biofilm measurements were normalized to remove plate-specific effects: for each plate the ratio (*r-plate*) between the mean of the wild-type measurements on the plate and the mean of all wild-type measurements was calculated. The normalized measurements were obtained by dividing the plate's biofilm measurements with the ratio *r-plate*. The Mann–Whitney *U*-test was used to compare each of the 13 mutant strains with the wild-type strain. Bonferroni correction was used to address the problem of multiple comparisons.

### Protein preparation and Western blot

Cultures were grown in BHI until OD = 0.8, in conditions as indicated, and 25 ml of culture was pelleted by centrifugation (11000 r.p.m., 2 min, 4°C) and resuspended in 600 μl of buffer A [200 mM KCl, 50 mM Tris-HCl (pH = 8), 1 mM EDTA, 10% glycerol]. Bacteria were disrupted using a bead-beater for 75 s. Samples were centrifuged for 5 min at 4°C, 14 000 r.p.m and the supernatant was transferred to a microcentrifuge tube. This fraction was centrifuged for an additional 20 min, 4°C, 14 000 r.p.m and the supernatant was transferred to a new tube and the protein concentration was determined using Bio-Rad protein assay, according to the manufacturer. Twelve micrograms of protein was separated on SDS-polyacrylamide gels at appropriate concentrations and transferred to PVDF membranes using a wet transfer apparatus. Membranes were blocked in 5% dry milk, 4°C overnight. Primary antibody (anti-Hfq, anti-Lmo0799 and anti-sigB) was diluted 1:5000 in PBST, primary antibody anti-actA was diluted 1:4000, and were incubated at RT for 3 h. Membranes were washed in PBST at RT 2 × 5 min, 2 × 15 min, 2 × 5 min before secondary antibody, anti-rabbit HRP-conjugated antibody, was diluted 1:3000 in PBST and incubated for 2 h at RT. Membranes were washed as described above, developed using the ECL+ Western blotting kit (Amersham) and visualized using the STORM apparatus (Molecular Dynamics). For the SDS protein extraction Western blot experiments: Protein was extracted by resuspending the pellet from 10 ml of culture in 400 μl of 1× SDS sample buffer (50 mM Tris-Cl pH 6.8, 5% beta-mercaptoethanol, 2% SDS, 0.1% bromophenol blue, 10% glycerol), bead-beating the solution for 75 s and thereafter centrifuging the solution at 14000 r.p.m. for 1 min. Twenty-five microlitres of the supernatant was separated by SDS-PAGE.

### Transposon library construction and screening

A transposon library was constructed using a plasmid carrying a mariner-based transposon (pMC39), as previously described (Cao *et al*., 2007). Approximately 8000 mutants were plated on motility agar at RT, and subsequently screened for lack of ring formation by visual inspection after 7 days. Detailed information can be found in *Supporting information* (Supporting Experimental Procedures).

### Luciferase assay

The pPL2luxP_help_ plasmid (Riedel *et al*., 2007) was inserted into *L. monocytogenes* EGDe by conjugation as described previously (Flamm *et al*., 1984). Three microlitres of overnight culture of EGDe pPL2luxPhelp was spotted on the surface of a low-agar BHI plate and allowed to grow at RT with alternating light. Luciferase expression was measured with an Xenogen/Calipher IVIS® Spectrum (2 min exposure, binning factor 4). Carbon source additives were dissolved in water to a final concentration of 1 M, of which 1 μl was spotted on the surface of the colony 30 min prior to measurement.

### Viable count measurement in rings

Bacteria were spotted on 0.3% agar containing plates and exposed to 10 cycles of 12 h light/12 h darkness. Approximately seven microlitres of agar was excised from the agar plate at indicated position (translucent or opaque ring, 2- or 11-day-old rings) and the content of each punctuation was resuspended in 1 ml of PBS before serial dilution and plating.

### Hydrogen peroxide assay

Bacteria were spotted on 0.3% agar containing plates and exposed to 10 cycles of 12 h light/12 h darkness. Bacteria from 2-day-old rings were excised by a Pasteur-pipette (∼ 7 μl) and resuspended in 1 ml of PBS before addition of H_2_O_2_ (60 mM final concentration). After 90 min of incubation (if not stated differentially) bacteria were diluted and spread on agar plates for viable counting.

### Catalase activity scoring

Bacteria were spotted on 0.3% agar containing plates and exposed to 10 cycles of 12 h light/12 h darkness. Ten microlitres of 1 M H_2_O_2_ was spread from the inner to the outer part of the colony (i.e. from the origin of inoculum to the outer edge of the colony – vertical to the translucent and opaque rings). By camera, the perpendicular amount of oxygen produced was scored by the formation of spheres in translucent and opaque rings during 1 min. The percentage of oxygen sphere production was calculated for opaque and translucent rings as the fraction of all spheres formed (100%) and plotted in Fig. 3B. The entire procedure can be followed in the Supplemental Movie 1.

## References

[b1] Akbar S, Gaidenko TA, Kang CM, O'Reilly M, Devine KM, Price CW (2001). New family of regulators in the environmental signaling pathway which activates the general stress transcription factor sigma(B) of *Bacillus subtilis*. J Bacteriol.

[b2] Aravind L, Koonin EV (2000). The STAS domain – a link between anion transporters and antisigma-factor antagonists. Curr Biol.

[b1001] Arnaud M, Chastanet A, Débarbouillé M (2004). New vector for efficient allelic replacement in naturally nontransformable, low-GC-content, gram-positive bacteria. Appl Environ Microbiol.

[b3] Avila-Perez M, Hellingwerf KJ, Kort R (2006). Blue light activates the sigmaB-dependent stress response of *Bacillus subtilis* via YtvA. J Bacteriol.

[b4] Avila-Perez M, Vreede J, Tang Y, Bende O, Losi A, Gartner W, Hellingwerf K (2009). *In vivo* mutational analysis of YtvA from *Bacillus subtilis*: mechanism of light activation of the general stress response. J Biol Chem.

[b6] Berleman JE, Kirby JR (2009). Deciphering the hunting strategy of a bacterial wolfpack. FEMS Microbiol Rev.

[b5] Berleman JE, Chumley T, Cheung P, Kirby JR (2006). Rippling is a predatory behavior in *Myxococcus xanthus*. J Bacteriol.

[b7] Bruno JC, Freitag NE (2011). *Listeria monocytogenes* adapts to long-term stationary phase survival without compromising bacterial virulence. FEMS Microbiol Lett.

[b8] Camilli A, Bassler BL (2006). Bacterial small-molecule signaling pathways. Science.

[b9] Cao M, Bitar A, Marquis H (2007). A mariner-based transposition system for *Listeria monocytogenes*. Appl Environ Microbiol.

[b10] Chan RH, Lewis JW, Bogomolni RA (2012). Photocycle of the LOV-STAS protein from the pathogen *Listeria monocytogenes*. Photochem Photobiol.

[b11] Crosson S, Rajagopal S, Moffat K (2003). The LOV domain family: photoresponsive signaling modules coupled to diverse output domains. Biochemistry.

[b12] Donati AJ, Jeon JM, Sangurdekar D, So JS, Chang WS (2011). Genome-wide transcriptional and physiological responses of *Bradyrhizobium japonicum* to paraquat-mediated oxidative stress. Appl Environ Microbiol.

[b13] Enos-Berlage JL, McCarter LL (2000). Relation of capsular polysaccharide production and colonial cell organization to colony morphology in *Vibrio parahaemolyticus*. J Bacteriol.

[b14] Ferreira A, O'Byrne CP, Boor KJ (2001). Role of sigma(B) in heat, ethanol, acid, and oxidative stress resistance and during carbon starvation in *Listeria monocytogenes*. Appl Environ Microbiol.

[b15] Flamm RK, Hinrichs DJ, Thomashow MF (1984). Introduction of pAM beta 1 into *Listeria monocytogenes* by conjugation and homology between native *L. monocytogenes* plasmids. Infect Immun.

[b16] Flemming HC, Wingender J (2010). The biofilm matrix. Nat Rev Microbiol.

[b17] Freitag NE, Port GC, Miner MD (2009). *Listeria monocytogenes* – from saprophyte to intracellular pathogen. Nat Rev Microbiol.

[b18] Friedman L, Kolter R (2004). Genes involved in matrix formation in *Pseudomonas aeruginosa* PA14 biofilms. Mol Microbiol.

[b19] Gaidenko TA, Kim TJ, Weigel AL, Brody MS, Price CW (2006). The blue-light receptor YtvA acts in the environmental stress signaling pathway of *Bacillus subtilis*. J Bacteriol.

[b20] Gorski L, Palumbo JD, Nguyen KD (2004). Strain-specific differences in the attachment of *Listeria monocytogenes* to alfalfa sprouts. J Food Prot.

[b21] Hain T, Hossain H, Chatterjee SS, Machata S, Volk U, Wagner S (2008). Temporal transcriptomic analysis of the *Listeria monocytogenes* EGD-e sigmaB regulon. BMC Microbiol.

[b22] Hamon M, Bierne H, Cossart P (2006). *Listeria monocytogenes*: a multifaceted model. Nat Rev Microbiol.

[b23] Harvey J, Keenan K, Gilmour A (2007). Assessing biofilm formation by *Listeria monocytogenes* strains. Food Microbiol.

[b24] Hecker M, Pane-Farre J, Volker U (2007). SigB-dependent general stress response in *Bacillus subtilis* and related gram-positive bacteria. Annu Rev Microbiol.

[b25] Lamb JS, Zoltowski BD, Pabit SA, Li L, Crane BR, Pollack L (2009). Illuminating solution responses of a LOV domain protein with photocoupled small-angle X-ray scattering. J Mol Biol.

[b26] Lemon KP, Freitag NE, Kolter R (2010). The virulence regulator PrfA promotes biofilm formation by *Listeria monocytogenes*. J Bacteriol.

[b27] Loh E, Dussurget O, Gripenland J, Vaitkevicius K, Tiensuu T, Mandin P (2009). A trans-acting riboswitch controls expression of the virulence regulator PrfA in *Listeria monocytogenes*. Cell.

[b28] Lopez D, Vlamakis H, Kolter R (2010). Biofilms. Cold Spring Harb Perspect Biol.

[b29] Losi A, Quest B, Gartner W (2003). Listening to the blue: the time-resolved thermodynamics of the bacterial blue-light receptor YtvA and its isolated LOV domain. Photochem Photobiol Sci.

[b30] Marvasi M, Visscher PT, Martinez LC (2010). Exopolymeric substances (EPS) from *Bacillus subtilis*: polymers and genes encoding their synthesis. FEMS Microbiol Lett.

[b31] Morgenstein RM, Szostek B, Rather PN (2010). Regulation of gene expression during swarmer cell differentiation in *Proteus mirabilis*. FEMS Microbiol Rev.

[b32] Nakano S, Kuster-Schock E, Grossman AD, Zuber P (2003). Spx-dependent global transcriptional control is induced by thiol-specific oxidative stress in *Bacillus subtilis*. Proc Natl Acad Sci USA.

[b33] O'Byrne CP, Karatzas KA (2008). The role of sigma B (sigma B) in the stress adaptations of *Listeria monocytogenes*: overlaps between stress adaptation and virulence. Adv Appl Microbiol.

[b34] Ondrusch N, Kreft J (2011). Blue and red light modulates SigB-dependent gene transcription, swimming motility and invasiveness in *Listeria monocytogenes*. PLoS ONE.

[b35] Premaratne RJ, Lin WJ, Johnson EA (1991). Development of an improved chemically defined minimal medium for *Listeria monocytogenes*. Appl Environ Microbiol.

[b36] Purcell EB, McDonald CA, Palfey BA, Crosson S (2010). An analysis of the solution structure and signaling mechanism of LovK, a sensor histidine kinase integrating light and redox signals. Biochemistry.

[b37] Rauprich O, Matsushita M, Weijer CJ, Siegert F, Esipov SE, Shapiro JA (1996). Periodic phenomena in *Proteus mirabilis* swarm colony development. J Bacteriol.

[b38] Riedel CU, Monk IR, Casey PG, Morrissey D, O'Sullivan GC, Tangney M (1993). Improved luciferase tagging system for *Listeria monocytogenes* allows real-time monitoring *in vivo* and *in vitro*. Appl Environ Microbiol.

[b39] Selby CP, Sancar A (2007). Molecular mechanism of transcription-repair coupling. Science.

[b40] Shimkets LJ, Kaiser D (1982). Induction of coordinated movement of *Myxococcus xanthus* cells. J Bacteriol.

[b41] Solano C, Garcia B, Valle J, Berasain C, Ghigo JM, Gamazo C, Lasa I (2002). Genetic analysis of *Salmonella enteritidis* biofilm formation: critical role of cellulose. Mol Microbiol.

[b42] Toledo-Arana A, Dussurget O, Nikitas G, Sesto N, Guet-Revillet H, Balestrino D (2009). The *Listeria* transcriptional landscape from saprophytism to virulence. Nature.

[b43] Truglio JJ, Croteau DL, Houten BV, Kisker C (2006). Prokaryotic nucleotide excision repair: the UvrABC system. Chem Rev.

[b44] van der Veen S, Abee T (2010). Importance of SigB for *Listeria monocytogenes* static and continuous-flow biofilm formation and disinfectant resistance. Appl Environ Microbiol.

[b45] Verstraeten N, Braeken K, Debkumari B, Fauvart M, Fransaer J, Vermant J, Michiels J (2008). Living on a surface: swarming and biofilm formation. Trends Microbiol.

[b46] Ziegelhoffer EC, Donohue TJ (2009). Bacterial responses to photo-oxidative stress. Nat Rev Microbiol.

[b47] Zuber P (2009). Management of oxidative stress in *Bacillus*. Annu Rev Microbiol.

