# Supplementary material for: Cycles of light and dark co-ordinate reversible colony differentiation in Listeria monocytogenes
Source: Mol Microbiol. 2013 Jan 21;87(4):909–24. doi: 10.1111/mmi.12140 (PMC3610012; doi:10.1111/mmi.12140)
Supplement: Supplementary file 1 [file mmi0087-0909-sd1.zip › mmi12140-sup-0001-si/mmi_12140_sm.pdf]

Cycles of light and dark coordinate reversible colony differentiation in *Listeria monocytogenes*

Supporting Information

Teresa Tiensuu[1,2,*], Christopher Andersson[1,2,*], Patrik Rydén[3,4] and Jörgen Johansson[1,2,#]

[1]Department of Molecular Biology, [2]Laboratory for Molecular Infection Medicine Sweden (MIMS)

[3]Department of Mathematics and Mathematical Statistics, [4]Computational Life science Cluster (CLiC)

Umeå University, 90187 Umeå, Sweden

[#]Corresponding author: E-mail: jorgen.johansson@molbiol.umu.se

Tel: +46-90-7852535

Fax: +46-90-772630

*Equal contribution

Supporting Experimental Procedures

Construction of the Δlmo0799 deletion mutant

A Δ*lmo0799* precise deletion mutant was generated by homologous recombination as previously described for *Listeria monocytogenes* Arnaud et al. 2004. In brief, two PCR products (using oligonucleotides KO0799A+KO0799B1 and KO0799C+KO0799D, Table S1), flanking *lmo0799*, were digested with the restriction endonucleases *Nco*I and *EcoR*I (for fragment KO0799A+KO0799B1) and *EcoR*I and *Sal*I (for fragment KO0799C+KO0799D). The digested fragments were ligated into corresponding sites in the pMAD suicide vector Arnaud et al. 2004, generating a pMAD plasmid containing the flanking regions of *lmo0799* but lacking the *lmo0799* coding region itself (+ 2 bases 5´and 3´ of *lmo0799*). This plasmid, pMADKO0799CD+AB1, was sequenced and used for homologous recombination and introduced into *Listeria monocytogenes* EGDe by electroporation. Transformants were selected on BHI plates containing erythromycin (7µg ml^{-1}) grown at 30°C. Isolated colonies were grown at 39°C in BHI with erythromycin (7µg ml^{-1}) over night. Serial dilutions were plated on BHI erythromycin (7 µg ml^{-1}) + X-gal (50 µg ml^{-1}) plates and grown in 41°C for two days. Blue colonies were selected and grown at 30°C for 6 hrs followed by incubation at 39°C for 3 hrs. Serial dilutions were plated on LA-X-gal (50 µg ml^{-1}) plates. White colonies were screened for erythromycin sensitivity and checked by PCR. Gene knock-out was verified by Northern blot.

Complementation of deletion mutant

A PCR fragment (oligonucleotides lysRb1 and lmo0799 U-Sal1) (Table S1), spanning approximately 130 bases upstream of the start codon of *lmo0799* to a region lying just downstream of the lysine riboswitch terminator, was digested with *EcoR*1 and *Sal*I restriction endonucleases and ligated (16° overnight) into the corresponding restriction sites in the plasmid

pMK4 (Table S1) using T4 DNA ligase (USB). The plasmid construct was sequenced and used to transform *Listeria monocytogenes* by electroporation (2.4 kV, 200Ω, 25µF). Transformed colonies were selected on Cml (7µg ml^{-1}) BHI plates and checked by PCR.

Transposon library construction and screening

Mutagenesis was conducted by introduction of the pMC39 plasmid into *L. monocytogenes* EGDe, as described by Cao et al. 2007. Briefly; the plasmid was inserted by electroporation (performed as described by Monk et al. 2008), and the electroporated cells were grown on BHI agar supplemented with 5µg ml^{-1} erythromycin (Em) at 30°C over night. Overnight culture of transformants were grown in BHI (10µg ml^{-1} Em and 10 µg ml^{-1} Kanamycin) at 30°C, diluted 1:200 into fresh BHI (10µg ml^{-1} Em) and grown at 30°C for 1h and then shifted to 40°C until an OD_{600} between 0,3 and 0,5 had been achieved. Culture aliquots were subsequently plated on BHI agar supplemented with 10µg ml^{-1} Em, and incubated at 40°C. Individual colonies were used to inoculate BHI (10µg ml^{-1} Em) in separate wells of 96-well plates, subsequently grown at 30°C until confluent. Glycerol was added to a final concentration of 15%, and the plates were stored at -80°C. Approximately 8000 transposon mutants were spotted onto low-agar (0,3% w/v) plates, allowed to grow at RT in alternating light on the bench (natural light oscillations) for 7 days, and screened for ring-formation by visual observation for a ring-less phenotype. Transposon insertion point was determined by two rounds of PCR. First primers Marq207/255 were used with colony lysate as template. The acquired PCR product was used as template for the second round of PCR using primers Marq208/256. The PCR product was subsequently sequenced using primer Marq257.

Complementation of transposon mutants:

PCR fragments corresponding to the genes *lmo2488, lmo0798, lmo2668, lmo0596* (oligonucleotides used for PCR see Table S2) were digested with the corresponding restriction enzymes. Fragments were ligated into digested plasmid pIMK3. The resulting constructs were sequenced and introduced into *E. coli* strain S17-1 either by electroporation (2.4 kV, 200 Ω, 25µF) or heat-shock treatment. Constructs were transferred to corresponding *Listeria monocytogenes* transposon mutant strains (tn::*lmo2488* (C14:E6), tn::*lmo0798* (C4:E12), tn::*lmo2668* (D2:C10), tn::*lmo0596* (C14:C12)) by conjugation (Simon et al. 1983). Conjugants were selected by plating on BHI plates containing kanamycin, colistin sulfate and nalidixic acid. The construct p*lmo0799* was used to complement the transposon mutants tn::*lmo0799* C13:D7, tn:: *lmo0799* C14:B5 and was introduced by electroporation (2.4 kV, 200 Ω, 25µF).

Supporting References:

Arnaud M, Chastanet A, Debarbouille M. 2004. New vector for efficient allelic replacement in naturally nontransformable, low-GC-content, gram-positive bacteria. *Appl Environ Microbiol* **70**: 6887-6891.

Brondsted L, Kallipolitis BH, Ingmer H, Knochel S. 2003. kdpE and a putative RsbQ homologue contribute to growth of *Listeria monocytogenes* at high osmolarity and low temperature. *FEMS Microbiol Lett* **219**: 233-239.

Cao M, Bitar AP, Marquis H. 2007. A mariner-based transposition system for *Listeria monocytogenes*. *Appl Environ Microbiol* **73**: 2758-2761.

Dramsi S, Levi S, Triller A, Cossart P. 1998. Entry of *Listeria monocytogenes* into neurons occurs by cell-to-cell spread: an in vitro study. *Infect Immun* **66**: 4461-4468.

Garsin DA, Urbach J, Huguet-Tapia JC, Peters JE, Ausubel FM. 2004. Construction of an Enterococcus faecalis Tn917-mediated-gene-disruption library offers insight into Tn917 insertion patterns. *J Bacteriol* **186**: 7280-7289.

Hain T, Hossain H, Chatterjee SS, Machata S, Volk U, Wagner S, Brors B, Haas S, Kuenne CT, Billion A et al. 2008. Temporal transcriptomic analysis of the *Listeria monocytogenes* EGD-e sigmaB regulon. *BMC Microbiol* **8**: 20.

Kocks C, Gouin E, Tabouret M, Berche P, Ohayon H, Cossart P. 1992. L. monocytogenes-induced actin assembly requires the actA gene product, a surface protein. *Cell* **68**: 521-531.

Kocks C, Hellio R, Gounon P, Ohayon H, Cossart P. 1993. Polarized distribution of Listeria monocytogenes surface protein ActA at the site of directional actin assembly. *J Cell Sci* **105 (Pt 3)**: 699-710.

Mackaness GB. 1964. The Immunological Basis of Acquired Cellular Resistance. *J Exp Med* **120**: 105-120.

Mengaud J, Dramsi S, Gouin E, Vazquez-Boland JA, Milon G, Cossart P. 1991. Pleiotropic control of Listeria monocytogenes virulence factors by a gene that is autoregulated. *Mol Microbiol* **5**: 2273-2283.

Monk IR, Gahan CG, Hill C. 2008. Tools for functional postgenomic analysis of *listeria monocytogenes*. *Appl Environ Microbiol* **74**: 3921-3934.

Riedel CU, Monk IR, Casey PG, Morrissey D, O'Sullivan GC, Tangney M, Hill C, Gahan CG. 2007. Improved luciferase tagging system for *Listeria monocytogenes* allows real-time monitoring in vivo and in vitro. *Appl Environ Microbiol* **73**: 3091-3094.

Simon R, Priefer U, Puhler A. 1983. A Broad Host Range Mobilization System for Invivo Genetic-Engineering - Transposon Mutagenesis in Gram-Negative Bacteria. *Bio-Technol* **1**: 784-791.

Sullivan MA, Yasbin RE, Young FE. 1984. New shuttle vectors for Bacillus subtilis and Escherichia coli which allow rapid detection of inserted fragments. *Gene* **29**: 21-26.

Toledo-Arana A, Dussurget O, Nikitas G, Sesto N, Guet-Revillet H, Balestrino D, Loh E, Gripenland J, Tiensuu T, Vaitkevicius K et al. 2009. The *Listeria* transcriptional landscape from saprophytism to virulence. *Nature* **459**: 950-956.

Table S1. **Transposon mutants deficient in ring-formation**

Mutant	Tn location	Function *	Membrane protein as predicted by sosui 1.11 **	Classification ***
A4:E8	lmo0040	conserved hypothetical protein	No	5.2 (From other organisms)
B12:G6	lmo0086	large hypothetical protein, 1959a.a. ******	Yes, 1 transmembrane helix	5.1 (From Listeria)
D9:B6	lmo0101	Highly similar to ArsR family transcriptional regulator****	No	3.5.2 (Regulation)
A4:D7	Between lmo0101 and lmo0102	Lmo0101: similar to transcriptional regulator, ArsR family. Lmo0102: similar to antibiotic biosynthesis monooxygenase****	No	Intergenic region
C10:A8	lmo0124	Unknown protein	No	6 (No similarity)
D2:G8	lmo0214	transcription-repair coupling factor, (superfamily II, involved in DNA restriction/modification and repair), mfd	No	3.2 (DNA restriction/modification and repair)
D16:C7	lmo0230	Uvr domain containing protein****	No	5.2 (From other organisms)
C1:B3	lmo0327	Similar to internalin****	Yes, 2 transmembrane helices	1.8 (Cell surface proteins)
D3:G4	lmo0333	InlJ, internalin protein, LPXTG motif	Yes, 2 transmembrane helices	1.8 (Cell surface proteins)
D15:G9	lmo0349	Hypothetical protein	Yes, 4 transmembrane helices	5.1 (From Listeria)
C4:H12	lmo0537	similar to N-carbamyl-L-amino acid amidohydrolase	No	2.2 (Metabolism of amino acids and related molecules)
A4:E5	lmo0544	similar to PTS system, glucitol/sorbitol-specific enzyme II CII component	Yes, 3 transmembrane helices	1.2 (Transport/binding proteins and lipoproteins)
C14:C12	Region between lmo0595/lmo0596	Lmo0595: similar to O-acetylhomoserine sulfhydrylase. Lmo0596:Unknown function	Lmo0596: Yes, 6 transmembrane helices. Lmo0595: No	Intergenic region
D4:E11	lmo0724	similar to B. subtilis YvpB protein	Yes, 1 transmembrane helix	5.2 (From other organisms)
C12:F3	lmo0774	Highly similar to diacylglycerol kinase domain-containing protein****	No	5.2 (From other organisms)
C4:E12	In between lmo0799 and lmo0798 (in the lysine riboswitch)	Lmo0798: similar to lysine-specific permease. Lmo0799: Light-receptor***	Lmo0798: Yes, 12 transmembrane helices. Lmo0799: No	Intergenic region
C14:B5	lmo0799	Light receptor*****	No	5.2 (From other organisms)
D15:E12	lmo0818	similar to cation transporting ATPase	Yes, 10 transmembrane helices	1.2 (Transport/binding proteins and lipoproteins)
A1:D10	lmo0842	putative peptidoglycan bound protein (LPXTG motif)	Yes, 2 transmembrane helices	1.8 (Cell surface proteins)
C14:A10	In front of lmo0887	Lmo0887 is a putative toxin for a MazF/PemK family Toxin-Antitoxin system****	No	Intergenic region
C14:A11	In front of lmo0887	Lmo0887 is a putative toxin for a MazF/PemK family Toxin-Antitoxin system****	No	Intergenic region
C8:F1	lmo0887	Putative toxin for a MazF/PemK family Toxin-Antitoxin system****	No	5.2 (From other organisms)
C8:G12	In between lmo0887/lmo0888	lmo0887/lmo0888 encodes a an endoribonuclease of the MazF/PemK family and its inhibitor******	No	Intergenic region
C1:C10	lmo0890	highly similar to negative regulation of sigma-B activity, rsbS	Yes, 2 transmembrane helices	4.1 (Adaptation to atypical conditions)
C1:C9	lmo0890	highly similar to negative regulation of sigma-B activity, rsbS	Yes, 2 transmembrane helices	4.1 (Adaptation to atypical conditions)
C10:F10	lmo0890	highly similar to negative regulation of sigma-B activity, rsbS	Yes, 2 transmembrane helices	4.1 (Adaptation to atypical conditions)
C4:F5	lmo0890	highly similar to negative regulation of sigma-B activity, rsbS	Yes, 2 transmembrane helices	4.1 (Adaptation to atypical conditions)
B5:B2	lmo0892	highly similar to serine phosphatase RsbU	No	4.1 (Adaptation to atypical conditions)
C4:D12	lmo0892	highly similar to serine phosphatase RsbU	No	4.1 (Adaptation to atypical conditions)
C11:C6	lmo0892	highly similar to serine phosphatase RsbU	No	4.1 (Adaptation to atypical conditions)
C18:F5	lmo0892	highly similar to serine phosphatase RsbU	No	4.1 (Adaptation to atypical conditions)
B4:E10	lmo0893	anti-anti-sigma factor (antagonist of RsbW), rsbV	No	4.1 (Adaptation to atypical conditions)
C19:H1	lmo0893	anti-anti-sigma factor (antagonist of RsbW), rsbV	No	4.1 (Adaptation to atypical conditions)
D2:F8	lmo0894	sigma-B activity negative regulator, RsbW	No	4.1 (Adaptation to atypical conditions)
C13:H7	lmo0957	similar to glucosamine-6-Phoasphate isomerase	No	2.1.1 (Specific pathways)
C18:A3	lmo1337	similar to B. subtilis yqgP, Rhomboid family protein	Yes, 7 transmembrane helices	5.2 (From other organisms)
C9:C1	lmo1736	Similar to acetyltransferase****	No	5.2 (From other organisms)
B8:C1	lmo1811	similar to ATP-dependent DNA helicase recG.	No	3.3 (DNA recombination)
B4:H9	lmo1969	similar to 2-keto-3-deoxygluconate-6-phosphate aldolase	No	2.1.1 (Specific pathways)
A5:B3	lmo2074	similar to unknown proteins	Yes, 1 transmembrane helix	5.2 (From other organisms)
A4:H9	lmo2094	similar to L-fuculose-phosphate aldolase	No	2.1.1 (Specific pathways)
B5:G9	lmo2109	similar to hydrolase	No	2.1.1 (Specific pathways)
D4:G8	lmo2464	similar to transcription regulator, tetR family****	No	3.5.2 (Regulation)
C14:E6	lmo2488	excinuclease ABC (subunit A), UvrA	No	3.2 (DNA restriction/modification and repair)
D2:C10	lmo2668	Similar to transcriptional antiterminator (BglG family)	No	3.5.2 (Regulation)
A4:B1	lmo2682	highly similar to potassium-transporting ATPase A chain, kdpA	Yes, 11 transmembrane helices	1.2 (Transport/binding proteins and lipoproteins)
A3:G10	lmo2777	similar to efflux protein	Yes, 11 transmembrane helices	1.2 (Transport/binding proteins and lipoproteins)
D9:B10	lmo2818	similar to transmembrane efflux protein	Yes, 13 transmembrane helices	1.2 (Transport/binding proteins and lipoproteins)

*(Predicted) function according to Listilist ("http://genolist.pasteur.fr/ListiList/"), unless otherwise specified
**http://bp.nuap.nagoya-u.ac.jp/sosui/
***Classifications according to Listilist "http://genolist.pasteur.fr/ListiList/"
****According to NCBI blastp "http://blast.ncbi.nlm.nih.gov/Blast.cgi"
*****Ondrusch and Kreft 2011
******Authors note

Table S2. Oligonucleotides used in this study

**Oligonucleotides used for Northern blotting (5´ to 3´)**	
lmo0263(inlH) fwd	CTGATCCAGCTCTTGCAATG
lmo0263(inlH) rev	ACTGCACTCCTTCTATCGTTG
lmo0596 fwd	GTTTCTGGCTGGGTACTAGC
lmo0596 rev	GCCAATTGTACGCATAATACC
lmo0676 fwd	GACGGGGTAATTCTAGTGTGG
lmo0676 rev	CCAAGCAGTAATAAGTGGTTGC
T7 lmo0676 fwd	TAATACGACTCACTATAGGACGGGGTTAATTGTAGTGTGG
lmo1830 fwd	CGAAGTGATTACTGCTGGTAG
lmo1830 rev	GAAAAAGTGGCGCTTCCAGTG
lmo2085 fwd	CTGGGGCACAACTAATCCAAC
lmo2085 rev	CCTGGGATTGTCCGAATATCC
lmo2573 fwd	CGATTTCCATCAATCCGGTAG
lmo2573 rev	CAAACCAACTAACCGCTCATC
lmo2673 fwd	GACGAGCTGTTCAATTTGCC
lmo2673 rev	GAAGGTAGTTTTTGGCGTACC
lmo2695 fwd	GGAAAAGTCGGACTTGTAAG
lmo2695 rev	CACCGGTATAGTTTTTCACG
lmo0799-U	CAACAGAGCACGACTATCAG
lmo0799-D	CTTCTTTGCCGTGATCCATG
lysRs- L	CATTTTGTCAGATAGCACACC
lysRs-R	GAATGAACAGGTTGATCGCC
lmo2230-U	GCATATTCGAAGTGCCATTGC
lmo2230-D	CAATGAGATCAGCATCTGCC
lmo0267-U(bsh)	GTTACGCCGAAAAATTACCCG
lmo0267-D(bsh)	CACATTGTCCTTACCTTCTGC
lmo1295-U (hfq)	CCCCCATGGGCATGAAACAAGGTGGACAAGGG
lmo1295-D (hfq)	CCCGGATCCTTATTCCGCATCAGGATTTAAAG
prfA-1	CTCGGAACCATATACTAACTC
prfA-2	GGGAAGGTTGATAACTTTCTCTTGCTTTAATTTGG
tmRNA-U	CGGCACTTAAATATCTACGAGC
tmRNA-L	CCTCGTTATCAACGTCAAAGCC
**Oligonucleotides used for *lmo0799* knock-out construction (5´ to 3´). Restriction enzyme site underlined.**	
KO 0799-A	GAAGATACTCCACACCTTCTATTG
KO 0799-B1	GGGGAATTCGCGTGTTTCTCCCCCTTTG
KO 0799-C	GGGGAATTCGATATTGCAAAATAGCCCGTTTTCC
KO 0799-D	GGGGTCGACGCTTTTACCGAAAGCGCATTTAAGC

**Oligonucleotides used for *lmo0799* complementation (5´ to 3´). Restriction enzyme site underlined.**		
lysRb1	GGGGAATTCCCTTTCTATTATCAGAGGAAGTG	
lmo0799 U-Sal1	GGGGTCGACCTTATCACCTCTAACTCC	
**Oligonucleotides used for the transposon library.**		
Name	Sequence	Reference
Marq 207	GGCCACGCGTCGACTAGTACNNNNNNNNNNG TAAT	Garsin et al. 2004
Marq 208	GGCCACGCGTCGACTAGTAC	Garsin et al. 2004
Marq 255	CAGTACAATCTGCTCTGATGCCGCATAGTT	Cao et al. 2007
Marq 256	TAGTTAAGCCAGCCCCGACACCCGCCAACA	Cao et al. 2007
Marq 257	CTTACAGACAAGCTGTGACCGTCT	Cao et al. 2007
**Oligonucleotides used for the complementation of Tn-mutants (5´ to 3´). Restriction enzyme site underlined.**		
Name	Sequence	
lmo0596up	AAAAAACTGCAGTTCGTAAAAATATGCATAAT	
lmo0596down	AAAAAAGGATCCATTAGAAAAAATAAAGGGGA	
uvrA- BamHI fwd	GGGGGATCCGGAAGGATGAAGCGAAATTGG	
uvrA-PstI-rev	GGGCTGCAGCTGTCATACAACCAGAGGATG	
lmo0798-BamHI-fwd	GGGGGATCCTAGAAAGGGAGATTTATAGTG	
lmo0798D-PstI	GGGCTGCAGCAAAACGCTCTACTGCAAACG	
lmo2668-BamHI fwd	GGGGGATCCGACAGGAGGGAACGATATG	
lmo2668-PstI rev	GGGCTGCAGTCTGACTTCTCTACATTGTC	

Table S3. Strains, plasmids and antibodies used in this study

**Strains**	**Reference**
Listeria monocytogenes	
EGDe (WT)	Mackaness 1964
Δ*sigB*	Brondsted et al. 2003
Δ*hfq*	Toledo-Arana et al. 2009
Δ*prfA*	Toledo-Arana et al. 2009
Δ*hly*	This work
Δ*actA*	This work
Δ*lmo0799*	This work
cz^{-}	This work
Listeria innocua	Dramsi et al. 1998
E. coli	
S17-1	Simon et al. 1983
**Plasmids**	
pMAD	Arnaud et al. 2004
pMK4	Sullivan et al. 1984
p*lmo0799*	This work
pMADKO0799CD+AB1	This work
pPL2*lux*Phelp	Riedel et al. 2007
pMC39	Cao et al. 2007
plis35	Mengaud et al. 1991
pactA-WT	Kocks et al. 1992
pIMK3	Monk et al. 2008
pIMK3::*lmo2488*	This work
pIMK3::*lmo0798*	This work
pIMK3::*lmo2668*	This work
pIMK3::*lmo0596*	This work
**Antibodies**	
Anti-Hfq	This work<br>(Antigen (NH_2)CTFSPQKNVALNPDAE$(CONH_2)$)
Anti-0799	This work<br>(Antigen<br>(NH_2)CKDVTTEHDYQLELEKSLTE$(CONH_2)$)
Anti-SigB	Hain et al. 2008
Anti-ActA	Kocks et al. 1993
Anti-rabbit-HRP	BioRad

Tiensuu_Supplemental Fig 1

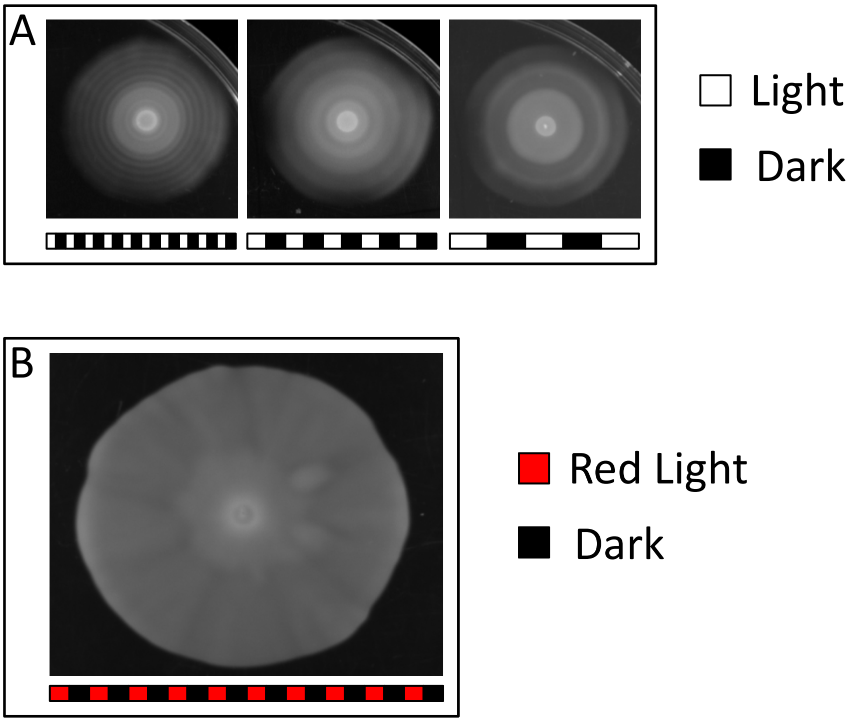

Figure S1. Bacterial phenotype on agar plates at different length of the light and dark cycles or exposure to red light. Upper panels: Wild-type *L. monocytogenes* were inoculated on low-agar plates and exposed to either 10 cycles of 6 hours blue light / 6 hours dark conditions (left panel), or 5 cycles of 12 hours blue light / 12 hours dark conditions (middle panel), or 2.5 cycles of 24 hours blue light / 24 hours dark conditions (right panel). Lower panel: Wild-type *L. monocytogenes* were inoculated on low-agar plates and exposed to 10 cycles of 12 hours red light / 12 hours dark conditions (light intensity 4 μm/m^2/sec).

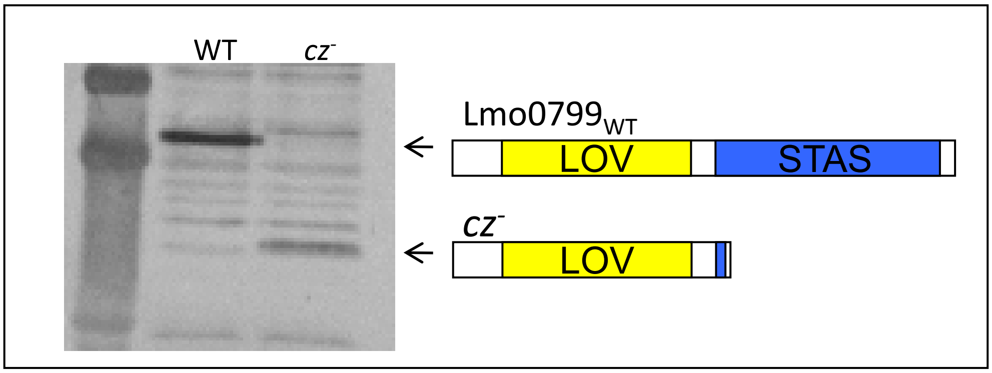

Figure S2. Western blot analysis of Lmo0799 protein. Expression of Lmo0799 in the wild-type (WT) and the consolidation negative *cz*⁻ strain as determined by western blot experiments. Full-length Lmo0799 contains a Light Oxygen Voltage domain (LOV, yellow) and an anti-anti sigma factor domain (STAS, blue).

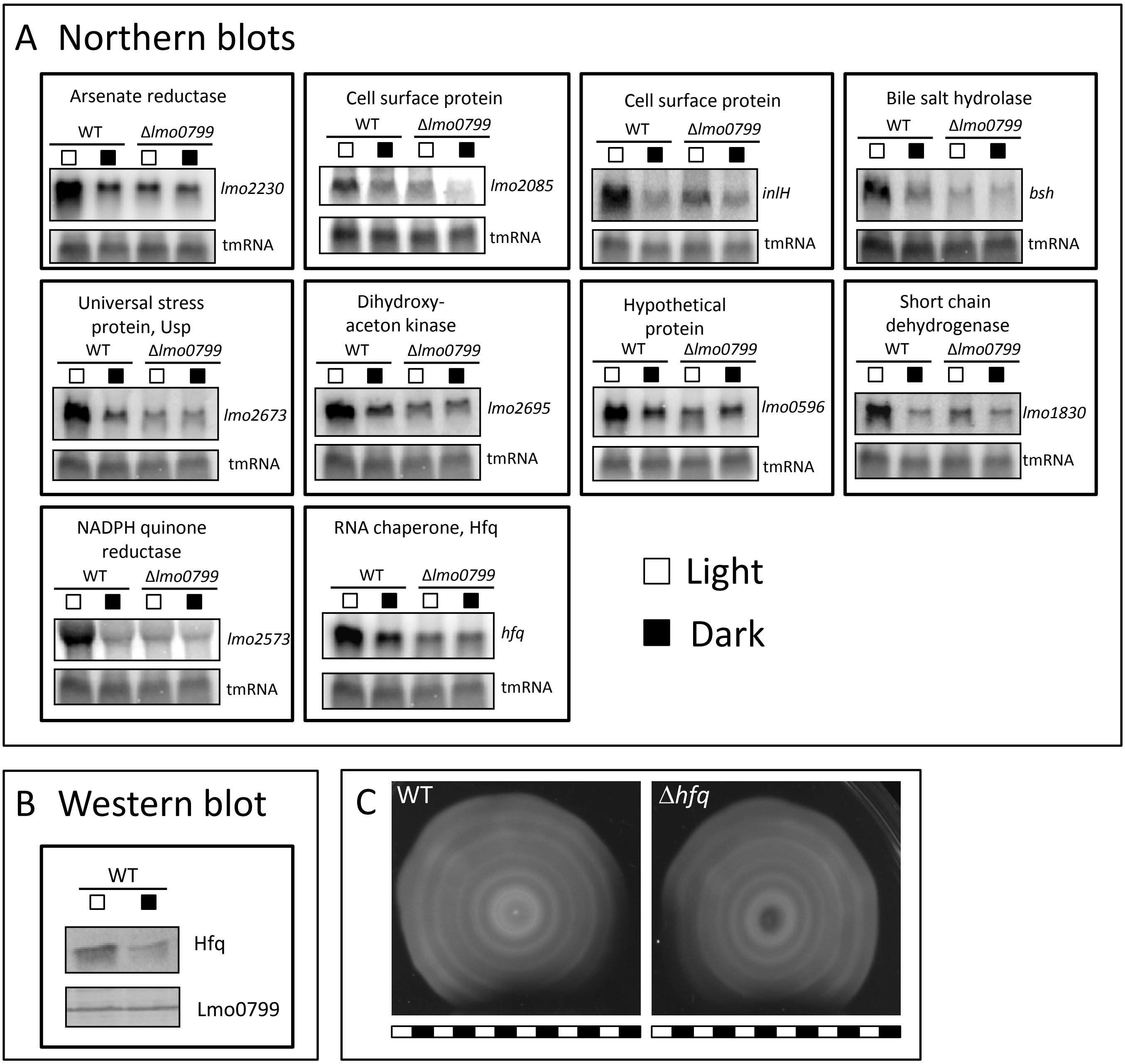

Figure S3. The blue-light receptor is required for light-induced expression of σ^B-regulated genes. A. Northern blot analysis of σ^B-regulated genes at light and dark conditions. Wild-type (WT) and Δ*lmo0799* strains were grown at light (open bar) or dark (black bar) conditions at 37°C, before RNA extraction and northern blot. The membranes were hybridized with *lmo2230*, *lmo2673*, *lmo0596*. *lmo2695*, *lmo2573*, *lmo1830*, *lmo2085*, *bsh*, *inlH*, *hfq* and tmRNA (control) specific DNA-probes. B. Western blot analysis of Hfq and Lmo0799 expression. Wild-type (WT) was grown at light or dark conditions 37°C, before protein extraction and western blot. The membranes were hybridized with α-Hfq or α-Lmo0799 antibodies before addition of a secondary antibody and development. C. Wild-type (WT) and Δ*hfq* strains were inoculated on low-agar plates and incubated for 6 cycles of 12 hours light / 12 hours dark conditions. An open bar indicates light conditions whereas a black bar indicates dark conditions.

Tiensuu_Supplemental Fig 4

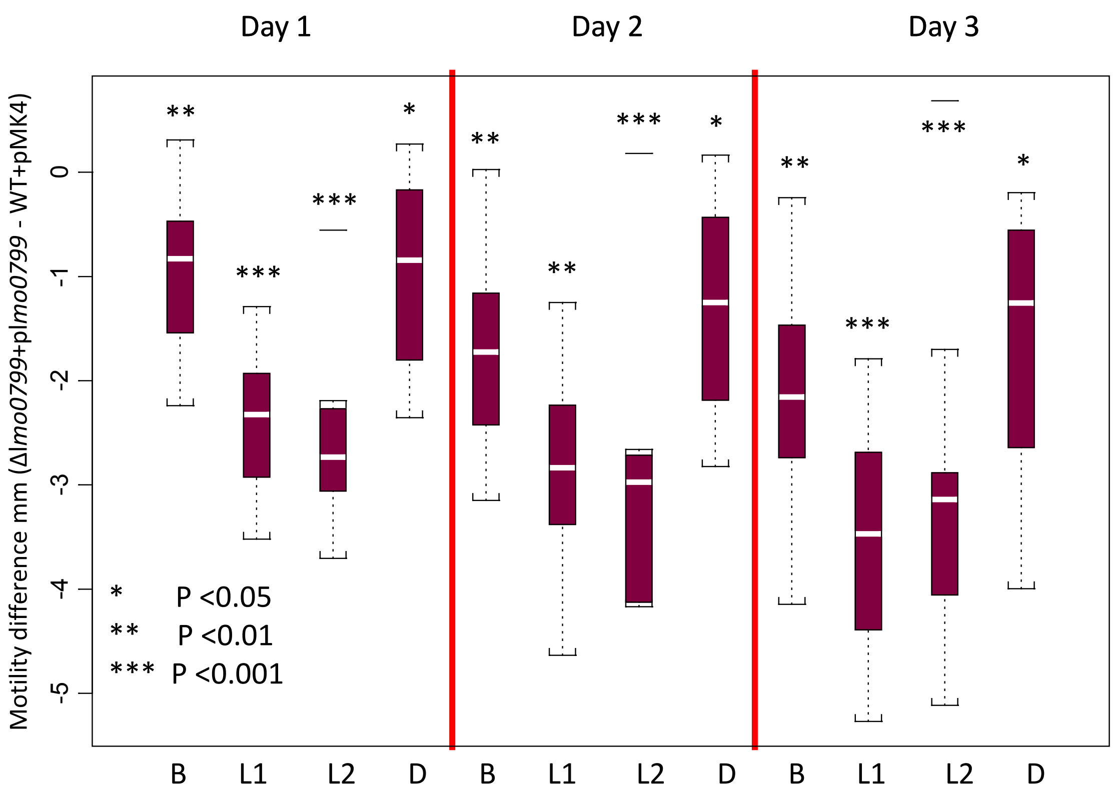

Figure S4. Lmo0799 inhibit *L. monocytogenes* motility at light conditions. Wild-type (WT) + pMK4 and Δ*lmo0799* + p*lmo0799* strains were inoculated on low-agar plates and incubated for indicated days on the bench (B); under blue-light enhanced aquarium light (L1); under laboratory light (L2) or at darkness (D). Bacterial motility was scored daily and the difference between the Δ*lmo0799* + p*lmo0799* and the WT + pMK4 was plotted.

Tiensuu_Supplemental Fig 5

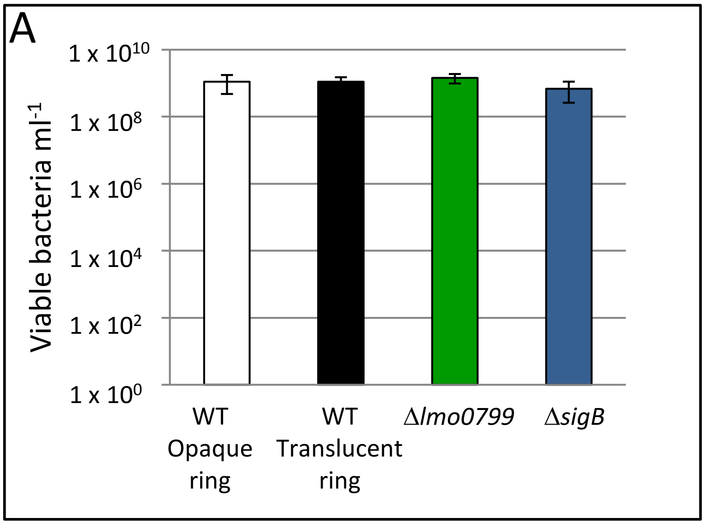

Figure S5. Bacteria in opaque and translucent rings show a similar amount of bacteria. Bacteria from WT, Δ*lmo0799* and Δ*sigB* strains were inoculated on a low-agar plate and exposed to 5 cycles of 12 hours light / 12 hours darkness before bacteria from 48 hour old opaque and translucent rings (for wild-type) or the corresponding region from the Δ*lmo0799* and the Δ*sigB* strains, were excised by punctuation using a Pasteur-pipette, resuspended and diluted **A.** The bacteria were plated on LA-agar plates incubated for 24 hours at 37°C before counting colonies. **B.** Diluted bacteria were Live/Dead stained and examined by confocal microscopy. Living bacteria show a green color whereas dead bacteria are stained red.

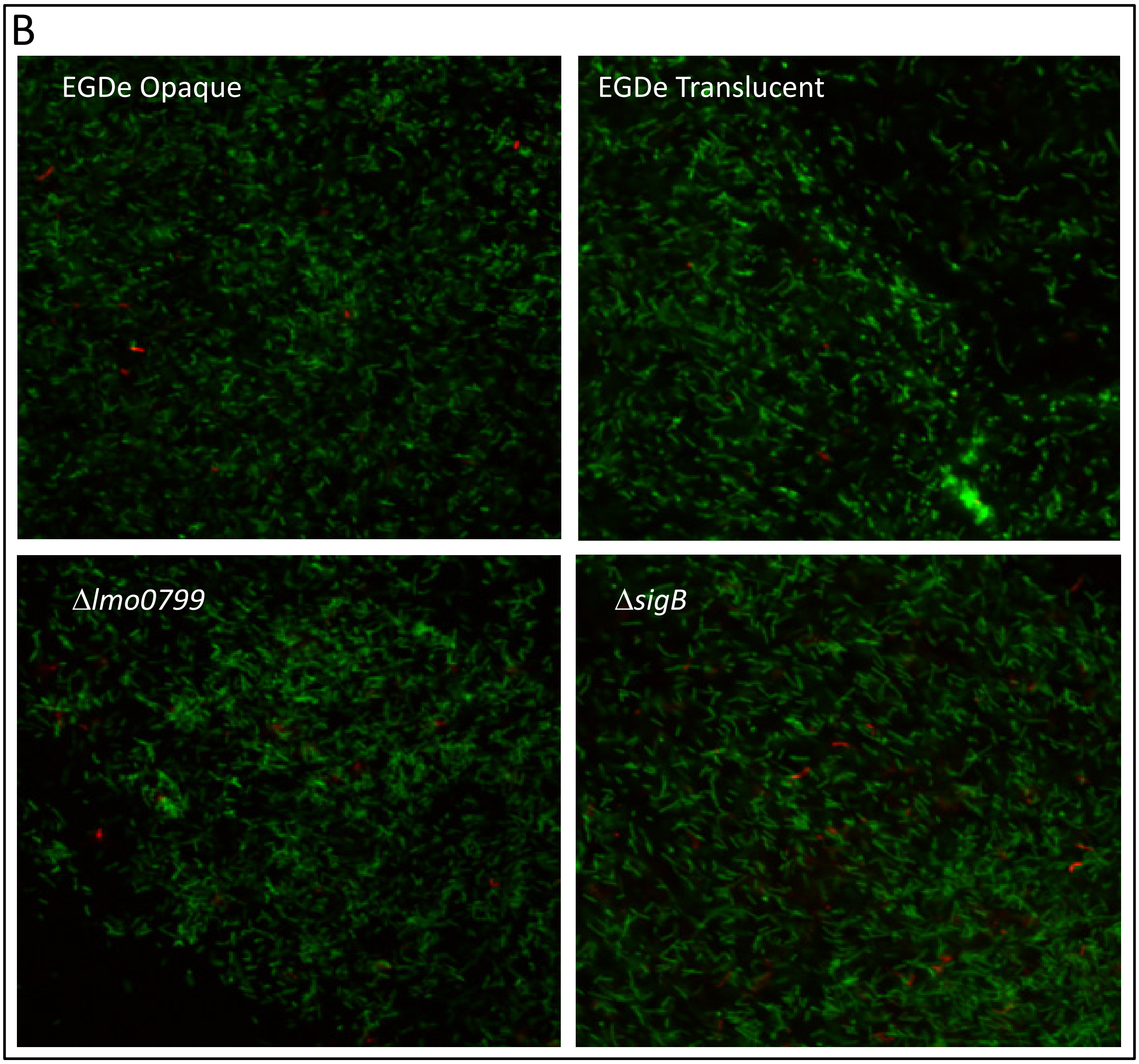

Tiensuu_Supplemental Fig 6

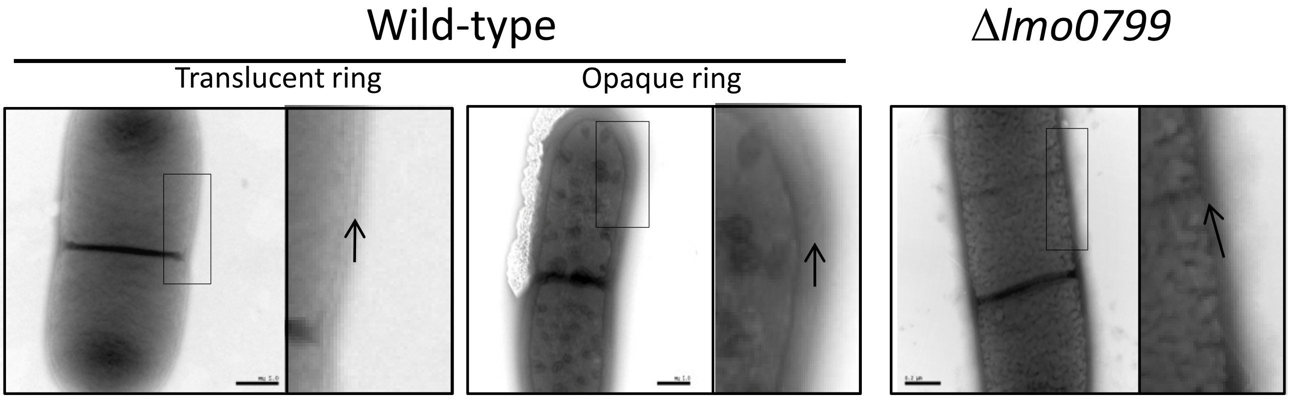

Figure S6. Cell-wall production in bacteria from opaque and translucent rings. WT and Δ*lmo0799* strains were inoculated on low-agar plates and exposed to 5 cycles of 12 hour light / 12 hour darkness before bacteria were excised from 48 hour old rings (or corresponding for the Δ*lmo0799* strain) stained and examined by Transmission Electron Microscopy. The bacterial cell-wall is highlighted by an arrow.

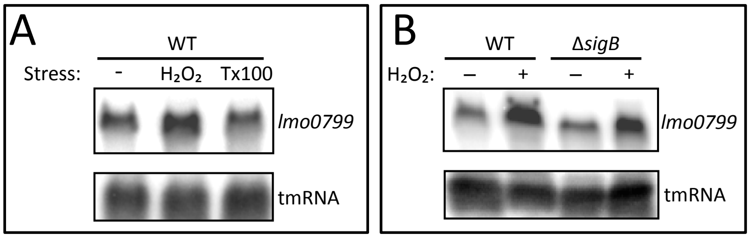

Figure S7. The gene encoding the blue-light receptor is induced by H_2O_2. A. Northern blot analysis of *lmo0799* expression at different stress-conditions. A wild-type (WT) *L. monocytogenes* strain was grown at 37°C until an OD_{600} = 0.8 in BHI prior to addition of H_2O_2 (H_2O_2) or TritonX-100 (Tx100) for 10 minutes. RNA was extracted and northern blots were performed. The membrane was hybridized with *lmo0799* and tmRNA (control) specific DNA-probes. B. Northern blot analysis of *lmo0799* expression in different strain backgrounds and at H_2O_2-stress. Wild-type (WT) and Δ*sigB* strains were grown at 37°C until an OD_{600} = 0.8 in BHI prior to addition of H_2O_2 for 10 minutes. RNA was extracted and northern blots were performed. The membrane was probed against *lmo0799* and tmRNA (control) specific DNA-probes.

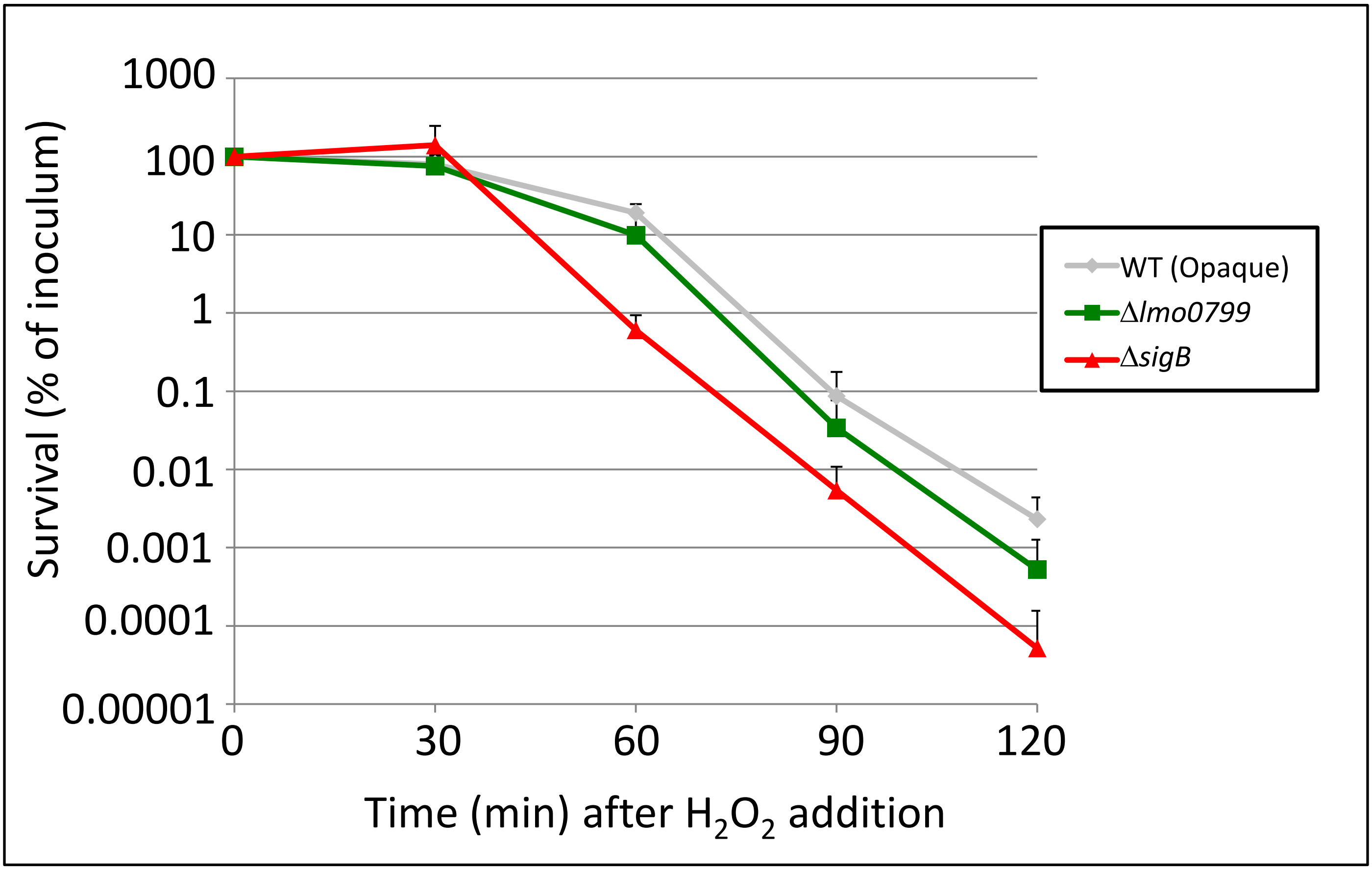

Figure S8. Lmo0799 and σ^B contributes to *L. monocytogenes* survival during H_2O_2-stress. WT, Δ*lmo0799* and Δ*sigB* strains were inoculated on low-agar plates and exposed to 8 cycles of 12 hours light / 12 hours darkness before bacteria were excised from 48 hour old opaque rings (or corresponding time-points for the Δ*lmo0799* and the Δ*sigB* strain), resuspended in 60 mM of H_2O_2 for up to 2 hours. Samples were removed, diluted and spread on agar plates at 0, 30, 60, 90 and 120 minutes after H_2O_2-addition, respectively (n=5).

Tiensuu_Supplemental Fig 9

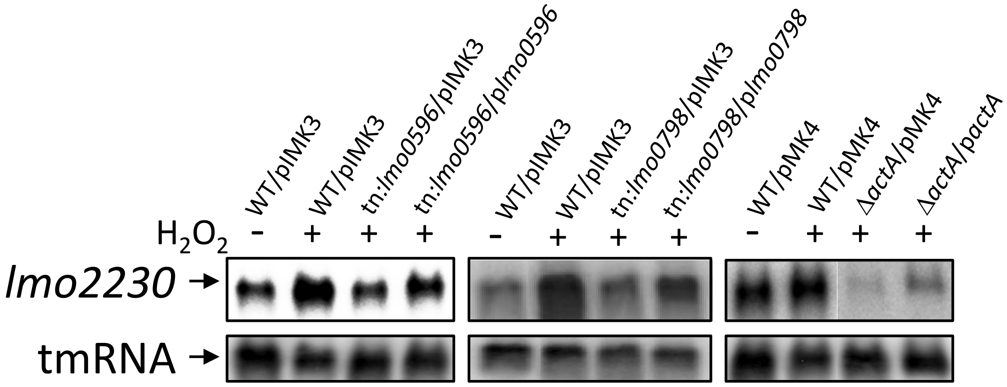

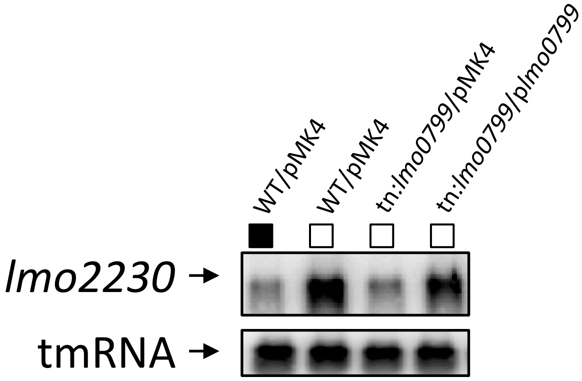

Figure S9. Restoring expression of genes inactivated by transposon mutants re-establish σ^B-activity. Indicated strains were grown in presence or absence of H_2O_2 (upper panels) or at dark/light conditions (lower panel) before RNA extraction and northern blot. The membrane was hybridized with *lmo2230* and tmRNA (control) specific DNA-probes.

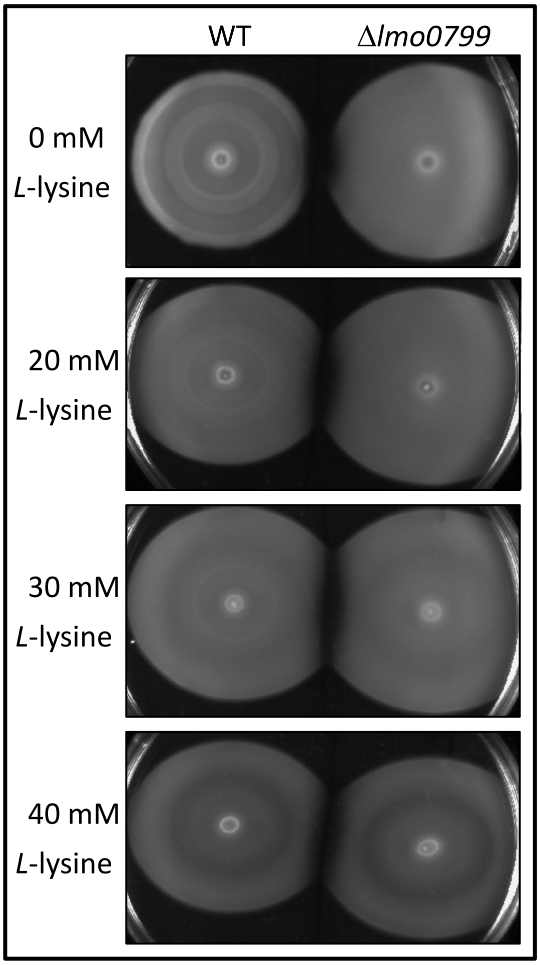

Figure S10. L-Lysine prevent ring-formation. Wild-type (WT) and Δ*lmo0799* strains were inoculated on low-agar plates containing 0 (-), 20, 30 or 40 mM L-lysine. Plates were exposed to 3 cycles of 24 hours light / 24 hours darkness.

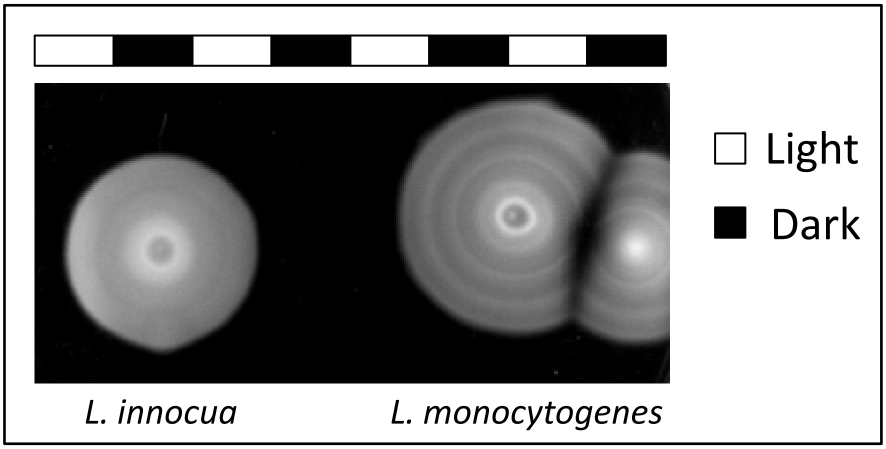

Figure S11. *Listeria innocua and L. monocytogenes* strains were inoculated on a low-agar plate and exposed to 4 cycles of 12 hours light / 12 hours darkness.

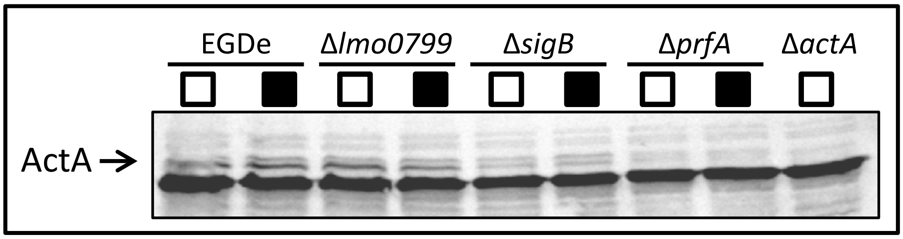

Figure S12. A. Western blot analysis of ActA expression. Indicated strains were grown in light (open box) or darkness (black box) before protein extraction and western blot. The membrane was hybridized with an α-ActA specific antibody.
